# NOX1 mediates metabolic heart disease in mice and is upregulated in monocytes of humans with diastolic dysfunction

**DOI:** 10.1093/cvr/cvab349

**Published:** 2021-11-26

**Authors:** Lifen Xu, Melania Balzarolo, Emma L Robinson, Vera Lorenz, Giacomo Della Verde, Lydia Joray, Michika Mochizuki, Beat A Kaufmann, Gideon Valstar, Saskia C A de Jager, Hester M den Ruijter, Stephane Heymans, Otmar Pfister, Gabriela M Kuster

**Affiliations:** Department of Biomedicine, University Hospital Basel and University of Basel, Hebelstrasse 20, 4031 Basel, Switzerland; Department of Biomedicine, University Hospital Basel and University of Basel, Hebelstrasse 20, 4031 Basel, Switzerland; Department of Cardiology, Cardiovascular Research Institute Maastricht, Maastricht University, Maastricht, The Netherlands; Department of Biomedicine, University Hospital Basel and University of Basel, Hebelstrasse 20, 4031 Basel, Switzerland; Department of Biomedicine, University Hospital Basel and University of Basel, Hebelstrasse 20, 4031 Basel, Switzerland; Department of Biomedicine, University Hospital Basel and University of Basel, Hebelstrasse 20, 4031 Basel, Switzerland; Department of Biomedicine, University Hospital Basel and University of Basel, Hebelstrasse 20, 4031 Basel, Switzerland; Department of Biomedicine, University Hospital Basel and University of Basel, Hebelstrasse 20, 4031 Basel, Switzerland; Department of Cardiology, University Hospital Basel, Basel, Switzerland; Department of Cardiology, Laboratory of Experimental Cardiology, University Medical Center Utrecht, Utrecht University, Utrecht, The Netherlands; Department of Epidemiology, Julius Center for Health Sciences and Primary Care, University Medical Center Utrecht, Utrecht University, Utrecht, The Netherlands; Department of Cardiology, Laboratory of Experimental Cardiology, University Medical Center Utrecht, Utrecht University, Utrecht, The Netherlands; Department of Cardiology, Laboratory of Experimental Cardiology, University Medical Center Utrecht, Utrecht University, Utrecht, The Netherlands; Department of Cardiovascular Sciences, Centre for Molecular and Vascular Biology, KU Leuven, Herestraat 49, Bus 911, 3000 Belgium, Leuven; Department of Cardiology, Maastricht University, CARIM School for Cardiovascular Diseases, Universiteitssingel 50, Maastricht 6229 ER, The Netherlands; ICIN-Netherlands Heart Institute, Holland Heart House, Moreelsepark 1, Utrecht 3511 EP, The Netherlands; Department of Biomedicine, University Hospital Basel and University of Basel, Hebelstrasse 20, 4031 Basel, Switzerland; Department of Cardiology, University Hospital Basel, Basel, Switzerland; Department of Biomedicine, University Hospital Basel and University of Basel, Hebelstrasse 20, 4031 Basel, Switzerland; Department of Cardiology, University Hospital Basel, Basel, Switzerland

**Keywords:** Diastolic dysfunction, Hypertrophy, Inflammation, Metabolic heart disease, NOX1

## Abstract

**Aims:**

Microvascular inflammation plays an important role in the pathogenesis of diastolic dysfunction (DD) and metabolic heart disease. NOX1 is expressed in vascular and immune cells and has been implicated in the vascular pathology of metabolic disease. However, its contribution to metabolic heart disease is less understood.

**Methods and results:**

NOX1-deficient mice (KO) and male wild-type (WT) littermates were fed a high-fat high-sucrose diet (HFHS) and injected streptozotocin (75 mg/kg i.p.) or control diet (CTD) and sodium citrate. Despite similar weight gain and increase in fasting blood glucose and insulin, only WT-HFHS but not KO-HFHS mice developed concentric cardiac hypertrophy and elevated left ventricular filling pressure. This was associated with increased endothelial adhesion molecule expression, accumulation of Mac-2-, IL-1β-, and NLRP3-positive cells and nitrosative stress in WT-HFHS but not KO-HFHS hearts. *Nox1* mRNA was solidly expressed in CD45^+^ immune cells isolated from healthy mouse hearts but was negligible in cardiac CD31^+^ endothelial cells. However, *in vitro, Nox1* expression increased in response to lipopolysaccharide (LPS) in endothelial cells and contributed to LPS-induced upregulation of *Icam-1*. *Nox1* was also upregulated in mouse bone marrow-derived macrophages in response to LPS. In peripheral monocytes from age- and sex-matched symptomatic patients with and without DD, *NOX1* was significantly higher in patients with DD compared to those without DD.

**Conclusions:**

NOX1 mediates endothelial activation and contributes to myocardial inflammation and remodelling in metabolic disease in mice. Given its high expression in monocytes of humans with DD, NOX1 may represent a potential target to mitigate heart disease associated with DD.

## Graphical Abstract

**Graphical Abstract:**
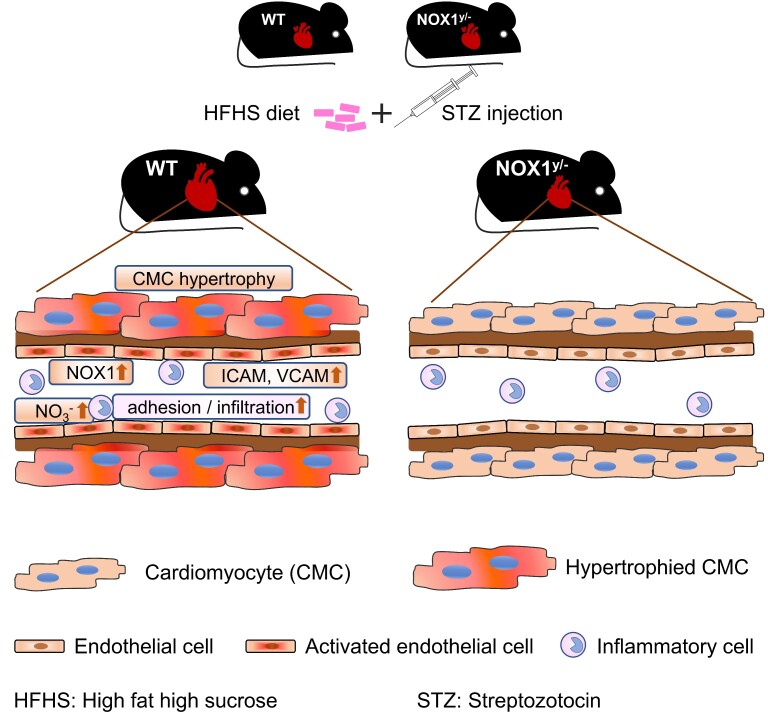

Translational perspective

In their multifactorial pathogenesis, diastolic dysfunction (DD) and heart failure with preserved ejection fraction (HFpEF) still remain poorly understood. They frequently occur in patients with obesity and metabolic syndrome. Microvascular inflammation and dysfunction have recently been recognized as major driving forces. We show that genetic deletion of *Nox1* prevents cardiac inflammation, remodelling, and dysfunction in metabolic disease in mice and find *NOX1* upregulated in peripheral monocytes of patients with DD. These findings add to our understanding how obesity, inflammation, and heart disease are linked, which is a pre-requisite to find therapeutic strategies beyond the control of co-morbidities in HFpEF.

## 1. Introduction

Obesity and metabolic syndrome are major health burdens in Western countries with reported 10–30% of individuals affected across Europe^1^ and >30% in the USA.^2^ A leading cause of death and morbidity in patients with metabolic syndrome are cardiovascular diseases.^3^ Besides coronary artery disease and hypertension, metabolic syndrome can induce a specific form of cardiomyopathy that occurs independently from macrovascular complications and is referred to as metabolic heart disease.^3,4^ Patients with metabolic heart disease may present with heart failure (HF) with reduced (HFrEF) or, more frequently, preserved ejection fraction (HFpEF).^4,5^ In the multifactorial pathogenesis of HFpEF, low-grade systemic inflammation elicited by obesity and diabetes^6^ leads to microvascular endothelial dysfunction, endothelial cell activation, and disturbed endothelial cell-cardiomyocyte crosstalk. This contributes to cardiomyocyte hypertrophy, stiffening, and impaired relaxation,^7,8^ which are hallmark features of the HFpEF phenotype of metabolic heart disease in humans.

NADPH oxidases (NOX) are transmembrane proteins that catalyze the production of reactive oxygen species (ROS), mostly superoxide, by transferring electrons from NADPH to molecular oxygen.^9^ The superoxide production by the isoforms NOX1 and 2 is inducible and depends on the agonist-dependent assembly of multiple subunits.^9^ NOX1 is expressed in endothelial^10,11^ and vascular smooth muscle cells^12,13^ and upregulated in diabetic arteries.^14^ Enhanced NOX activity reduces the bioavailability of nitric oxide (NO) through peroxynitrite formation and uncoupling of endothelial NO synthase (eNOS),^15–17^ which has specifically been shown for NOX1 in diabetic mouse aorta.^18^ NOX1 has also been implicated in the accumulation of macrophages in the aorta of diabetic and atherosclerosis-prone mice.^19^ In addition, NOX1 is expressed in mesenteric resistance arteries, where it contributes to microvascular dysfunction in metabolic disease in mice.^20^ However, little is known about the expression and function of NOX1 in the heart and its role in cardiac disease. We therefore sought to assess the role of NOX1 in metabolic heart disease in mice, and validated our findings in monocytes derived from patients with and without clinically defined diastolic dysfunction (DD).

## 2. Methods

An Expanded Methods section is available in the Supplementary material online.

### 2.1. Mice

NOX1-deficient mice were a kind gift from Dr Karl-Heinz Krause, University Hospitals of Geneva and University of Geneva, Switzerland, and have previously been described.^21^ Upon receipt, the mice were bred to C57Bl/6N mice (Charles River) for at least four generations before use. For all studies, hemizygous NOX1^y/−^ (NOX1 is located on the X chromosome) (KO) and male wild-type (WT) littermates were used (see Supplementary material online, *Figure S1* for genotyping). All animal procedures complied with the NIH Guide for the Care and Use of Laboratory Animals and were approved by the Swiss Cantonal Authorities (Licence number 28347).

### 2.2. Experimental mouse model of metabolic disease

Four-week-old mice were randomly assigned to receiving high-fat high-sucrose (HFHS) diet (D12331, Research Diets Inc., New Brunswick, USA; g%: 23 protein, 17.5 sucrose, 17 maltodextrin 10, 0 starch, 35.8 fat, 5.56 kcal/g) (HFHS) or being continued on regular chow (3436, KLIBA NAFAG, Kaiseraugst, Switzerland; g%: 18.5 protein, 35 starch, 4.5 fat, 3.84 kcal/g) (control diet, CTD). After 3 weeks on diet, HFHS mice were injected a single dose of streptozotocin (STZ, 75 mg/kg; i.p.) diluted in sodium citrate (vehicle, 20 mM in 0.9% saline, pH 4.5), and CTD mice were injected with vehicle only (Supplementary material online, *Figure S2*).

### 2.3. Echocardiography

Transthoracic echocardiography was performed prior to STZ/vehicle injection and at 12, 24, 36, and 44 weeks after injection using a Vevo 2100 high-resolution small animal digital ultrasound system (VisualSonics) equipped with a linear-array transducer (MS550) operating at a centreline frequency of 40 MHz. Echocardiography (five sessions per animal in total) was performed in light anaesthesia using isoflurane (5% for induction, 2% for maintenance). After removal of the chest hair using a depilatory agent (Nair^TM^), mice were placed on a heated platform in the supine position. Body temperature was kept around 37°C. Left parasternal short-axis views at the mid papillary muscle level of the left ventricle (LV) and 2D-guided M-mode images were used to measure LV internal diameter at end-diastole and end-systole (LVID; d and LVID; s) and LV anterior wall (LVAW) and posterior wall (LVPW) thicknesses. LV mass was calculated based on the corrected cube formula (1.053 × [(LVID; d + LVPW; d + LVAW; d)^3^—LVID; d^3^] × 0.8). LV volumes in diastole (according to Teichholz: LV Vol; d = (7.0/(2.4 + LVID; d)) × LVID; d^3^) and systole (LV Vol; s = (7.0/(2.4 + LVID; s)) × LVID; s^3^) were used to calculate the left ventricular ejection fraction (LVEF) as follows: LVEF = 100 × (LV Vol; d—LV Vol; s)/LV Vol; d. For the evaluation of LV diastolic function, the transmitral inflow velocities were recorded with pulsed wave Doppler in the apical four-chamber view and mitral annular velocity was assessed with tissue Doppler imaging with the sample volume placed at the base of the posterior wall in a parasternal long axis. Thus, the measured and calculated Doppler indexes included the ratio of peak velocity of early to late filling of mitral inflow (*E*/*A*), the early diastolic mitral annulus velocity (*E*′), the ratio of *E* to *E*′ (*E*/*E*′), ejection time (ET), isovolumic contraction time (IVCT) and isovolumic relaxation time (IVRT). The LV myocardial performance index (Tei index) was calculated as (IVCT + IVRT)/ET. In addition, speckle tracking echocardiography was used to assess diastolic longitudinal peak strain rate [reverse longitudinal strain rate (rLSR)] at 44 weeks. For this purpose, the endocardium was manually traced on a parasternal long-axis view. Manual adjustments were done when needed. Endocardial strain values were then calculated with the strain package (Vevo Strain Package, Vevo 2100 version 1.6.0). All parameters were measured in three consecutive cardiac cycles and values were averaged. All data were acquired and analysed by one investigator blinded for animal treatment allocation.

### 2.4. Haemodynamic measurements

Invasive haemodynamic measurements were performed 44 weeks after STZ/vehicle injection. Mice were anaesthetized with urethane (1200 mg/kg) mixed with alpha-chloralose (50 mg/kg) i.p., intubated via the orotracheal route and ventilated on a Harvard Apparatus HSE Mouse Ventilator MiniVent 845. A jugular vein was cannulated for administration of fluid and a pressure volume (PV) catheter (1.0F, PVR-1035, ADInstruments, Houston, USA) was inserted through the carotid artery. Heart rate and arterial blood pressure were continuously monitored. The catheter was then advanced into the LV and pancuronium (2 mg/mL; 50 µl i.p.) was applied for final recording of the PV-loops to avoid breathing artefacts. After recording of steady-state measurements, LV preload was reduced by pressure on the inferior caval vein and load-independent parameters of contractility were measured (ML870 Powerlab 8/30, ADInstruments; MPVS Ultra, Millar Instruments). Recordings were then analysed offline in a blinded fashion using LabChart 7 Pro (ADInstruments).

At the end of invasive haemodynamic measurements, 10 IU heparin was infused through the jugular vein just before maximum blood collection (∼1 mL) from the right ventricle for volume calibration. The heart was arrested in diastole with 35 mmol/L KCl in PBS infused through the jugular vein, followed by perfusion fixation with 15 ml 4% paraformaldehyde (PFA) at a pressure between 80 and 100 mm Hg. The heart was excised and fixed in 4% PBS buffered formaldehyde (Polysciences, Inc., Warrington, USA) at 4°C for 24 h before further processing and embedding in paraffin. Mice that were not subjected to haemodynamic assessment were sacrificed directly after the last echocardiography by infusion of 35 mmol/L KCl/PBS through the jugular vein under deep anaesthesia with constant exposure to 5% isoflurane flow. The heart was perfused and fixed the same way as described above. Mice used for cell analyses were euthanized by i.p. injection of 200 mg/kg sodium pentobarbital.

### 2.5. Patients

Gene expression analysis was performed on flow-sorted CD14^+^ monocytes in a case–control selection of 20 individuals from the HELPFul study conducted at the University Medical Center (Universitair Medisch Centrum) Utrecht, the Netherlands. This single-centre, prospective, case-cohort study has been previously described.^22^ Patients aged 45 years and older, without previous cardiac interventions or congenital heart disease, that were referred by the general practitioner for diagnostic work-up to the cardiologist and gave written informed consent were eligible for inclusion. Patients were deemed as having DD based on the algorithm for LV DD of the HFA-PEFF score described by Pieske *et al.*^23^ and on current guidelines.^24^ The study protocol was approved by the Institutional Review Board of the University Medical Center Utrecht and complied with the principles outlined in the Declaration of Helsinki in October 2013. Trial registration NTR6016; Pre-results.^22^

### 2.6. Statistics

Data are shown in the figures as median and 10–90 percentile and listed in tables as mean ± SEM, unless indicated otherwise. Repeated measurements over time (echocardiography, weight) were tested for significance using a linear mixed effect model on R (version 3.4.1) and RStudio (1.0.153). Endpoint data were analysed with two-way ANOVA followed by Sidak *post hoc* test using Prism 9.2.0 (GraphPad Software, Inc.). Comparisons of two groups were done by Student’s *t*-test or non-parametric test as indicated. A *P*-value <0.05 was considered statistically significant.

### 2.7. Graphical abstract

The graphical abstract was drafted with PowerPoint using a cell shape for cardiomyocytes from Servier Medical Art (smart.servier.com).

## 3. Results

### 3.1. High-fat high-sucrose diet combined with a single low dose of streptozotocin induces metabolic disease in mice, independently of NOX1

To test the role of NOX1 in metabolic heart disease, we induced metabolic disease in WT and KO mice by HFHS and a single low-dose STZ injection (75 mg/kg i.p.). Wild-type and KO mice fed regular chow (CTD) and injected with vehicle (sodium citrate) were used as controls (Supplementary material online, *Figure S2*). Over 44 weeks from injection, WT-HFHS and KO-HFHS mice showed a continuous and comparable weight gain that was significantly greater than the one of WT-CTD and KO-CTD mice (Supplementary material online, *Figure S3A*). Compared to WT-CTD mice, WT-HFHS mice also showed a transient increase of fasting blood glucose, peaking around 8 weeks after STZ injection (Supplementary material online, *Figure S3B*), impaired glucose clearance in the glucose tolerance test (Supplementary material online, *Figure S3C*) and higher fasting plasma insulin levels (Supplementary material online, *Figure S3D*), and the same was true for KO-HFHS compared to KO-CTD mice. In summary, HFHS/STZ led to a metabolic disease phenotype in mice, independently of NOX1.

### 3.2. NOX1 promotes cardiac hypertrophy in mice with metabolic disease

**Figure 1:**
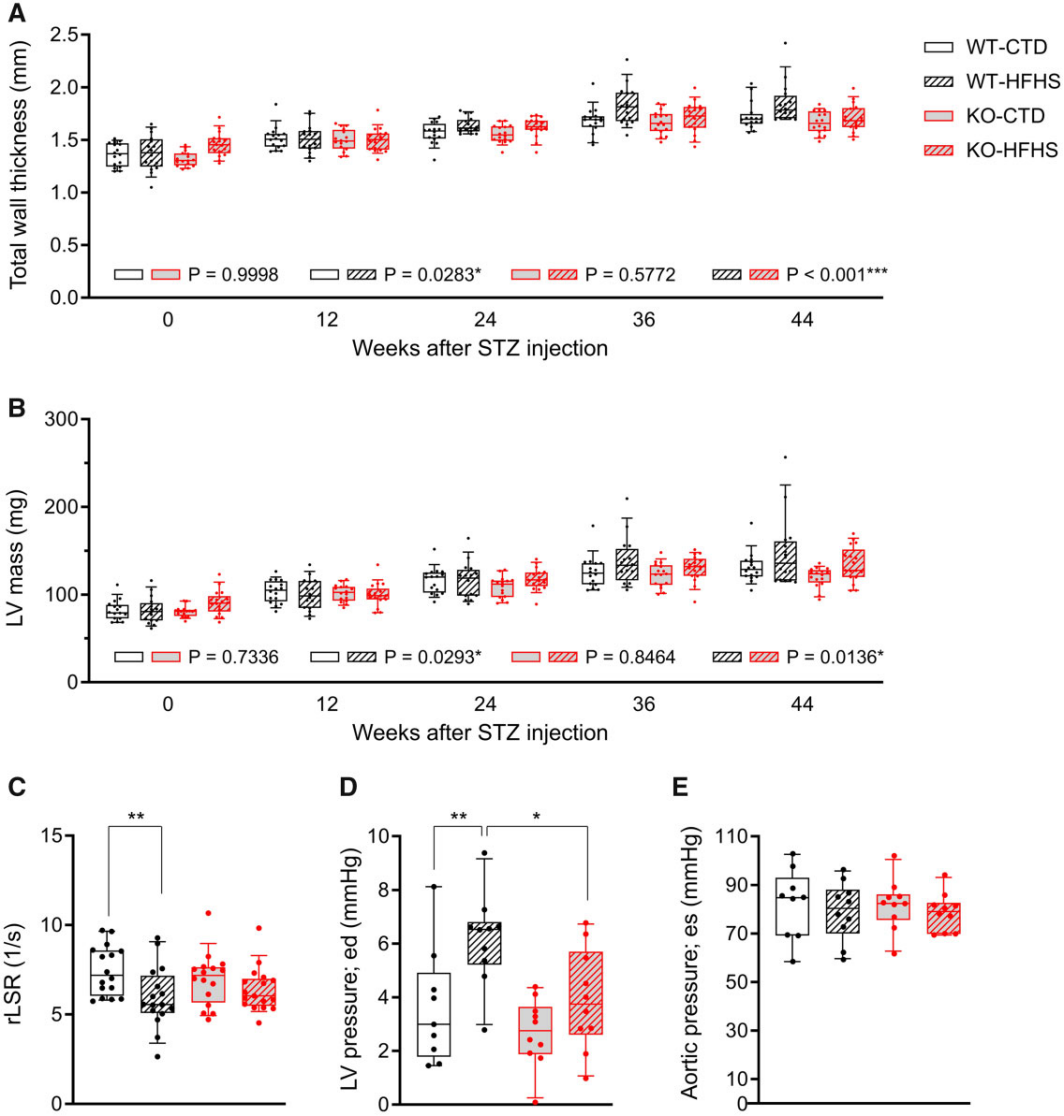
(*A, B*) Left ventricular structure assessed by echocardiography from the parasternal short axis view (M-mode). Mice were randomized to HFHS or CTD at 4 weeks of age and injected once with STZ or vehicle after 3 weeks on diet. Echocardiography was performed at 8- to 12-week intervals, comparisons between groups were done by fitting a R-Studio linear mixed effect model. (*n* = 16 for WT-CTD, 16 for WT-HFHS, 16 for KO-CTD, 17 for KO-HFHS) (*A*) Total wall thickness (LVAW; d + LVPW; d); (*B*) Left ventricular mass as calculated based on the corrected cube formula 1.053 × [(LVID; d; + LVPW; d + LVAW; d)^3^—LVID; d^3^] × 0.8. CTD, control diet; d, diastolic; HFHS, high-fat high-sucrose diet; LVAW, left ventricular anterior wall; LVID, left-ventricular internal diameter; LVPW, left ventricular posterior wall; STZ, streptozotocin. (*C–E*) Diastolic function and left-ventricular end-diastolic pressure. (*C*) Peak reverse longitudinal strain rate by echocardiography from the parasternal long-axis view (B-mode) in WT and KO mice at 44 weeks after STZ/vehicle injection and on respective diet (*n* = 16 for WT-CTD, 16 for WT-HFHS, 16 for KO-CTD, 17 for KO-HFHS); (*D*) Left ventricular end-diastolic pressures assessed by pressure-volume loop at 44 weeks; (*E*) Corresponding aortic end-systolic pressures as per *Figure 3D* (*D,E*: *n* = 9 for WT-CTD, 10 for WT-HFHS, 10 for KO-CTD, 10 for KO-HFHS). Groups were compared using two-way ANOVA followed by Sidak test, *P < 0.05, **P < 0.01. ed, end-diastolic; es: end-systolic; rLSR, reverse longitudinal strain rate.

The HFpEF phenotype of metabolic heart disease in humans includes LV hypertrophy associated with DD. Repeated echocardiography showed an increase in diastolic LV anterior (LVAW) and posterior wall (LVPW) thicknesses beginning week 36 after STZ injection in WT-HFHS mice, resulting in a significant increase in total wall thickness (calculated as LVAW+LVPW) compared to WT-CTD mice. This increase was not observed in KO-HFHS mice (Supplementary material online, *Table S1* and *Figure 1A*). Similarly, HFHS led to an increase in LV mass in WT, but not in KO mice (*Figure 1B*), whereas LV internal diameters were not different (Supplementary material online, *Table S1*). This indicates that genetic deletion of NOX1 prevents the development of concentric hypertrophy in our model.

Metabolic heart disease may be associated with HFpEF or HFrEF.^4^ We found no significant differences in LV ejection fraction over time within or between the groups (Supplementary material online, *Table S1*). Similarly, and despite the observed concentric hypertrophy in WT-HFHS mice, the *E*/*A* and *E*/*E*′ ratios were also not different (Supplementary material online, *Table S1*). In addition, no significant differences were found for ET, IVCT, IVRT, and Tei index (data not shown). However, the reverse longitudinal strain rate (rLSR), which has recently been identified as a sensitive parameter of impaired LV relaxation in mice,^25^ significantly decreased in WT-HFHS compared to WT-CTD, but not in KO-HFHS compared to KO-CTD mice (*Figure 1C*).

Because echocardiography has limited sensitivity for detection of elevated LV filling pressure in mice, a subset of consecutive mice was subjected to hemodynamic assessment prior to sacrifice. Forty-four weeks after STZ injection, WT-HFHS mice exhibited significantly elevated LV end-diastolic pressure (Ped) compared to WT-CTD mice. In contrast, Ped in KO-HFHS mice was comparable to that of KO-CTD mice and significantly lower than that of WT-HFHS mice (*Figure 1D*). Consistently, in comparison to WT-CTD, the maximum rate of LV pressure decay (d*P*/d*t* min), a measure of LV relaxation, was significantly decreased in WT-HFHS, but not in KO-HFHS mice compared to KO-CTD (Supplementary material online, *Table S2*). In turn, LV end-systolic pressure (Pes) was not different between groups, whereas the maximum rate of LV pressure rise (dP/dt max), a measure of global contractility, was decreased in WT-HFHS compared to WT-CTD mice, indicating some compromise of systolic function (Supplementary material online, *Table S2*).

Sustained blood pressure elevation can induce cardiac hypertrophy. At 44 weeks, aortic systolic pressure showed no significant difference between WT-HFHS and KO-HFHS mice (*Figure 1E*), and the same was true for mean blood pressure and pulse pressure, suggesting that the concentric hypertrophy occurs in the absence of sustained blood pressure elevation.

### 3.3. NOX1 contributes to increased heart weight, cardiomyocyte size, and nitrosative stress

**Figure 2:**
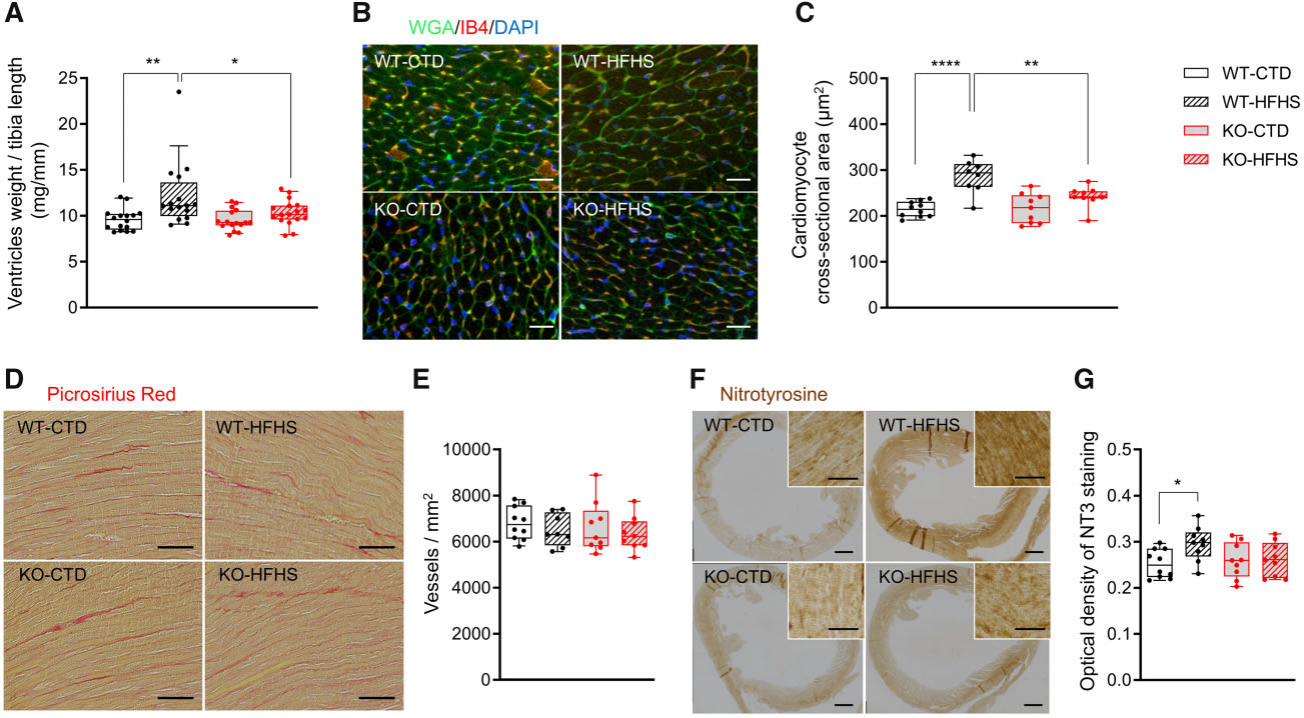
Heart weight, cardiomyocyte size, fibrosis, vessel density, and nitrotyrosinylation 44 weeks after STZ/vehicle injection. (*A*) Post-mortem heart weight normalized to tibia length (*n* = 16 for WT-CTD, 16 for WT-HFHS, 16 for KO-CTD, 17 for KO-HFHS); (*B*) Wheat germ agglutinin (WGA) and isolectin B (IB4) staining, scale bar: 20 μm; (*C*) Quantification of cardiomyocyte cross-sectional area based on WGA staining; (*D*) Representative microscope images from picrosirius red staining, scale bar: 50 μm; (*E*) Quantification of vessel density based on IB4 staining; (*F*) Representative nitrotyrosine staining of the myocardium, scale bar: 500 μm; insert scale bar: 20 μm. (*G*) Quantification of nitrosative stress based on nitrotyrosine staining (NT3). For quantifications, groups were compared using two-way ANOVA with Sidak test, *P < 0.05, **P < 0.01,****P < 0.0001; (*B–G*: *n* = 10 for WT-CTD, 8 in *B–E* and 9 in *F–G* for WT-HFHS, 9 for KO-CTD, 9 for KO-HFHS).

Forty-four weeks after STZ/vehicle injection, we found that the heart weight was significantly increased in WT-HFHS compared to WT-CTD mice. This increase was not observed in KO-HFHS mice, which showed a comparable heart weight to that of KO-CTD mice (*Figure 2A*). To examine whether the increase in heart weight was due to increased cardiomyocyte size, increased extracellular matrix deposition, or both, cross-sectional heart sections were stained with wheat germ agglutinin (WGA) and picrosirius red. WT-HFHS mice had a significantly higher average cardiomyocyte size than WT-CTD and KO-HFHS mice (*Figure 2B and C*). In contrast, the amount of fibrosis was not different between groups (*Figure 2D* and Supplementary material online, *Figure S4A–C*). Because myocardial fibrosis inversely correlates with microvascular density, we assessed myocardial microvascular density using isolectin B (IB4) staining. Consistent with the lack of enhanced fibrosis, we did not find differences in microvascular density between the groups (*Figure 2B and E*).

Superoxide, produced by intrinsic cardiac and immigrated inflammatory cells, can react with NO to form peroxynitrite, which exacerbates tissue damage and decreases NO bioavailability, and which can lead to nitration of protein tyrosine residues. We therefore assessed protein nitrotyrosinylation in the hearts after 44 weeks by immunohistochemistry. Immunoreactivity for nitrotyrosine was increased in the hearts of WT-HFHS as compared to WT-CTD, but not in KO-HFHS versus KO-CTD mice (*Figure 2F and G*).

### 3.4. NOX1 drives coronary endothelial activation and accumulation of inflammatory cells in the heart

**Figure 3:**
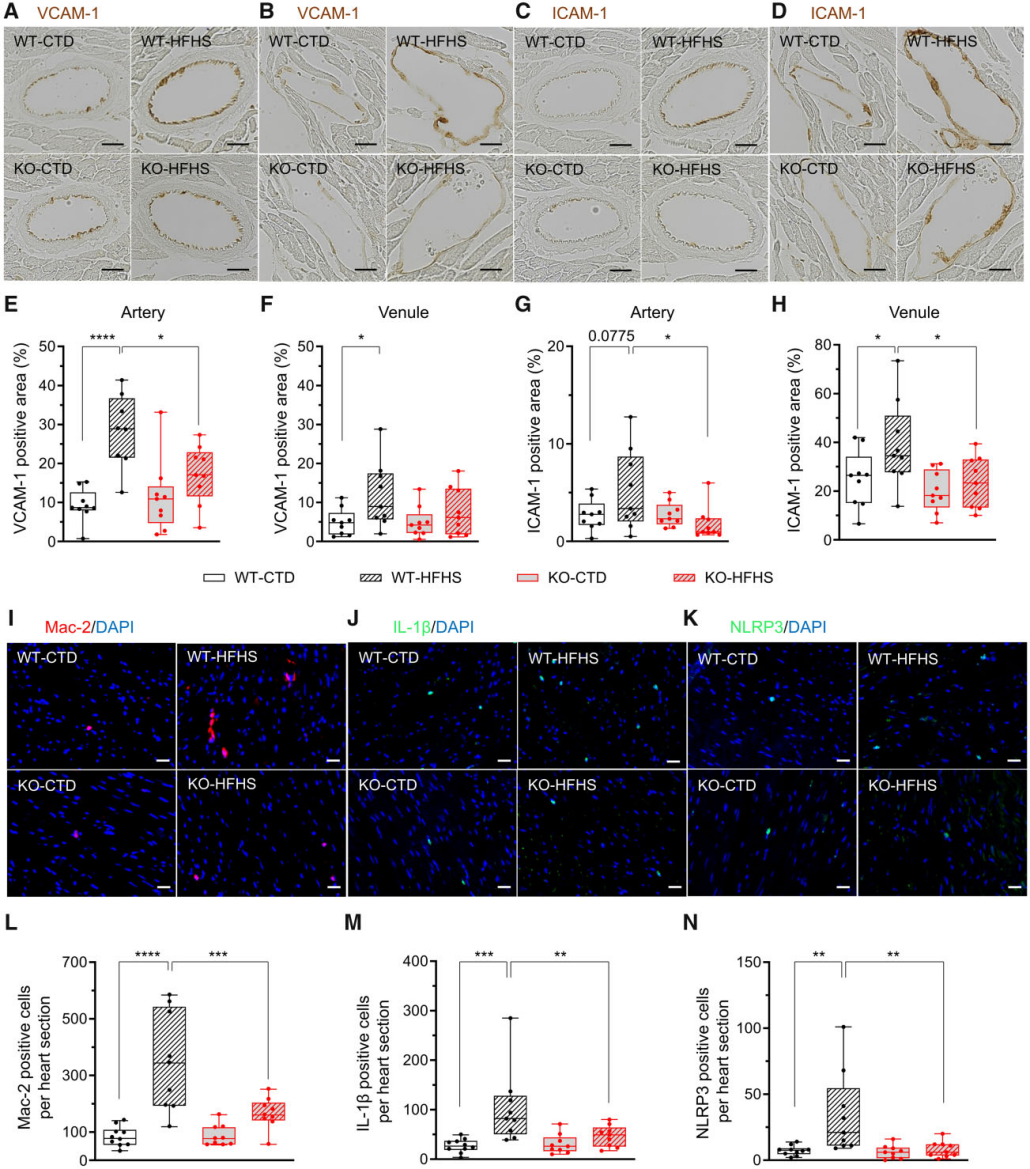
Endothelial activation based on VCAM-1 and ICAM-1 expression and cardiac inflammatory response by staining of Mac-2-, IL-1β-, and NLRP3 positive cells 44 weeks after STZ/vehicle injection. (*A–D*) Representative microscopic images for myocardial vessels stained with VCAM-1 and ICAM-1, scale bar: 20 μm. (*E–H*) Quantification of VCAM-1 and ICAM-1 expression in coronary arteries and venules. (*I–K*) Representative microscopic images for inflammatory cells stained with Mac-2, IL-1β, and NLRP3, scale bar: 20 μm; (*L–N*) Quantification of Mac-2-, IL-1β-, and NLRP3 positive cells per heart section. Groups were compared using two-way ANOVA with Sidak test, *P < 0.05, **P < 0.01, ***P < 0.001, ****P < 0.0001, (*n* = 10 for WT-CTD, 9 for WT-HFHS, 9 for KO-CTD, 9 for KO-HFHS).

Metabolic disease elicits chronic inflammation, characterized by increased expression of adhesion molecules in the vascular endothelium and accumulation of inflammatory cells in the tissue.^26^ We compared the expression of the vascular cell adhesion molecule 1 (VCAM-1) and intercellular adhesion molecule 1 (ICAM-1) on the endothelial surface of coronary arteries and venules in WT and KO mice under HFHS or CTD by immunohistochemistry. At 44 weeks after STZ/vehicle injection, WT-HFHS mice exhibited significantly enhanced VCAM-1 and ICAM-1 expression compared to WT-CTD and to KO-HFHS (*Figure 3A–H*). Similarly, the number of Mac-2 positive cells was significantly increased in WT-HFHS compared to WT-CTD and KO-HFHS mice (*Figure 3I and L*).

We also assessed whether the expression of the pro-inflammatory cytokine interleukin-1β (IL-1β), a key factor in the inflammation associated with obesity and metabolic syndrome,^27^ was modulated in the heart upon HFHS and by NOX1-deficiency. Circulating levels of IL-1β are increased in HFpEF^28^ and secretion of mature IL-1β depends on the activation of the NOD-like receptor family pyrin domain-containing 3 (NLRP3) inflammasome, which can be activated by NOX-derived ROS.^29,30^ We found significantly more IL-1β- (*Figure 3J and M*) and NLRP3-positive cells (*Figure 3K and*N**) in the hearts of WT-HFHS compared to both WT-CTD and KO-HFHS mice.

We further investigated whether the increased numbers of cells showing inflammatory activation in the hearts of WT-HFHS mice could result from the enhanced mobilization of immune cells to the peripheral blood. We did not find differences in the frequency of peripheral blood monocytes between WT-CTD and WT-HFHS mice at any of the analysed timepoints up to 24 weeks after STZ/vehicle injection. Monocyte frequencies in WT-HFHS and KO-HFHS were also comparable (Supplementary material online, *Figure S5A* and *B*). This suggests that the increase in myocardial tissue inflammation upon STZ/HFHS may not arise from differences in blood monocyte mobilization but may depend on other mechanisms that could be endothelium- and/or immune cell-mediated.

### 3.5. NOX1 can be induced in various cell types and contributes to adhesion molecule expression of endothelial cells

**Figure 4:**
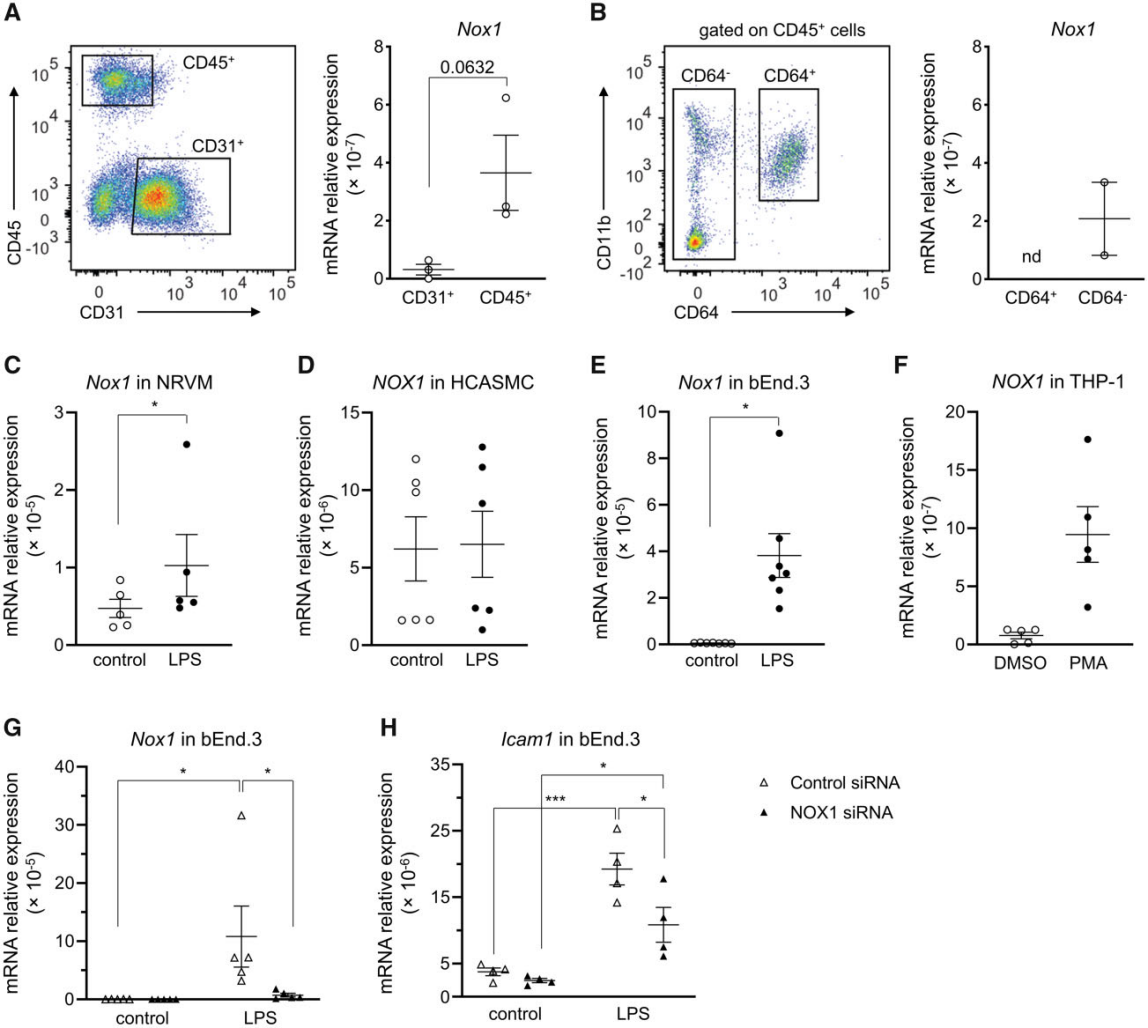
Nox1 expression in different cell types ex vivo and upon LPS or PMA stimulation in vitro. (*A*, left panel) Flow cytometry plot representing an example of the gating strategy used to sort CD31+ and CD45+ cells from WT mouse hearts. Full gating strategy is shown in Supplementary material online, *Figure S6*. (*A*, right panel) CD31+ and CD45+ cells were sorted from hearts of WT mice, and Nox1 mRNA expression was assessed by RT-qPCR. Each data point represents a pool of cells sorted from 5 to 6 mice, from three independently performed experiments. Data are shown as mean ± SEM. Comparison was made using unpaired *t*-test. (*B*, left panel) Flow cytometry plot representing an example of the gating strategy used to sort CD11b^+^CD64^+^ (CD64^+^) and CD64^-^ subpopulations from WT mouse hearts. (*B*, right panel) CD11b^+^CD64^+^ (CD64^+^) and CD64^-^ cells were sorted from WT mouse hearts and *Nox1* mRNA levels were assessed by RT-qPCR. Each data point represents a pool of five mice, from two independently performed experiments. nd, not detectable. Data are shown as mean ± SEM. (*C*–*F*) Expression of NOX1 mRNA in non-stimulated cells and after 6 h of stimulation with LPS or PMA in different cell types; bEnd.3, mouse endothelial cells; HCASMC, human coronary artery smooth muscle cells; NRVM, neonatal rat ventricular myocytes; THP-1, human monocyte cell line. Groups were compared using ratio-paired *t*-test for normally distributed data and Wilcoxon test for non-normally distributed data (HCASMC, bEnd.3, and THP-1 cells), *P < 0.05. (*G, H*) bEnd.3 cells were transfected with NOX1-targeting (NOX1 siRNA) or control siRNA and *Nox1* and *Icam1* mRNA expression were assessed in non-stimulated cells and after 6 h of LPS stimulation. Groups were compared using two-way ANOVA with Sidak test, *P < 0.05, ***P < 0.001.

Because NOX1 regulates endothelial and immune cell functions,^31,32^ we sorted CD45^+^ haematopoietic and CD31^+^ endothelial cells from the heart of WT mice and assessed *Nox1* mRNA expression (*Figure 4A* and Supplementary material online, *Figure S6*). *Nox1* mRNA was primarily expressed in CD45^+^ cells, whereas CD31^+^ cells showed undetectable to more than 5-fold lower levels of *Nox1* mRNA (*Figure 4B*). Given that NOX1 has been implicated in macrophage differentiation and function,^32,33^ we further analysed its level of expression in this cell lineage in the healthy heart. We sorted cardiac CD11b^+^CD64^+^ cells (Supplementary material online, *Figure S6*), which in the healthy murine heart contain cardiac macrophages^34,35^ and compared their *Nox1* mRNA levels to those of CD64^−^ cells, which comprise all remaining immune cells isolated from the heart. Whereas we could detect *Nox1* mRNA in CD64^−^ cells, *Nox1* mRNA was undetected in CD11b^+^CD64^+^ cells (*Figure 4C*). These observations suggest that in the healthy heart NOX1 is primarily expressed in immune cells other than cardiac macrophages.

We found increased expression of adhesion molecules on the endothelium of WT-HFHS, but not of KO-HFHS hearts. Therefore, even if not notably expressed in endothelial cells in the healthy heart, NOX1 could be upregulated in response to metabolic stress and this could also be the case in macrophages and other cell types represented in the heart. In addition, other isoforms of NOX, in particular NOX2, might also be regulated. In order to test this, we analysed *Nox1* and *Nox2* mRNA expression in neonatal rat ventricular myocytes (NRVM), human coronary artery smooth muscle cells (HCASMC), mouse bEnd.3 endothelial cells, and human monocytes (THP-1 cells) at baseline and upon exposure to lipopolysaccharide (LPS) or phorbol 12-myristate 13-acetate (PMA, THP-1 cells). *Nox1* was significantly upregulated in response to LPS in NRVM and bEnd.3 cells and in response to PMA in THP-1 cells, but not in LPS-stimulated HCASMC (*Figure 4C–F*). In contrast, *Nox2* mRNA could be induced in NRVM and THP-1, but not in bEnd.3 cells (Supplementary material online, *Figure S7A*–*C*). These findings support a NOX2-independent role of NOX1 in endothelial cells.

To further study the potential mechanism of NOX1-action in our model, we tested whether NOX1 was involved in the regulation of adhesion molecule expression in endothelial cells. We focused on ICAM-1, which was upregulated in cardiac arteries and venules of WT-HFHS compared to WT-CTD mice, but not of KO-HFHS versus KO-CTD mice. *Icam-1* mRNA expression was upregulated in bEnd.3 cells in response to LPS stimulation. This upregulation was significantly reduced upon knock-down of NOX1 through siRNA (*Figure 4G and H*).

### 3.6. NOX1 is upregulated in peripheral monocytes of humans with DD and induced by LPS in bone marrow-derived macrophages from mice

Because we found *NOX1* upregulated in response to PMA in human monocytes *in vitro*, we sought to further explore whether *NOX1* is regulated in human disease. We therefore sorted CD14^+^ monocytes from the peripheral blood of a small subset of patients with DD and of age- and sex-matched controls from the HELPFul cohort.^22^ Patient characteristics are given in Supplementary material online, *Table S3*. *NOX1* mRNA was robustly expressed in peripheral monocytes from all individuals but was significantly higher in patients with DD (Supplementary material online, *Figure S8A*), which also showed higher expression of *NOX2* expression in these cells (Supplementary material online, *Figure S7D*). In addition, *Nox1* mRNA was also detectable in bone marrow-derived macrophages (BMDMs) from mice and induced upon stimulation with LPS as previously also shown by others^36^ (Supplementary material online, *Figure S8B*).

## 4. Discussion

In a mouse model of metabolic disease, we demonstrate that NOX1 is mandatory for cardiac endothelial activation and myocardial remodelling. Lack of NOX1 correlates with a lower abundance of inflammatory cells in the heart of HFHS/STZ mice, and with less VCAM-1 and ICAM-1 expression in cardiac endothelial cells. We also find that NOX1 is upregulated in peripheral monocytes from patients with DD. These findings provide novel evidence for a central role of NOX1 in myocardial inflammation in metabolic disease, a condition frequently associated with DD in humans.

We induced metabolic disease through a combination of HFHS diet with an early single injection of low dose STZ, which resulted in a reproducible phenotype of metabolic disease featuring obesity and early signs of type II diabetes without the need for insulin treatment. This metabolic phenotype was similar in WT and KO mice regarding the degree of weight gain and glucose and insulin elevation. STZ/HFHS mice did not show an increase in systolic, diastolic, or mean arterial pressure as measured invasively after 44 weeks on diet, which is consistent with previous observations in diet-only, STZ, or genetic models of metabolic disease.^37–39^ Our findings therefore support the concept that arterial hypertension is not a pre-requisite for cardiac hypertrophy and also argue against large vessels being the exclusive site of NOX1 action in our model.

NOX1 contributes to macrovascular complications in diabetes and metabolic disease. In NOX1-deleted ApoE^−/−^ mice, atherosclerosis formation in the aorta upon STZ-induced diabetes was attenuated^40^ and this correlated with less ROS production and reduced macrophage infiltration.^19,40^ NOX1 also plays a role in metabolic microvascular disease. Introducing a genetic deletion of NOX1 in leptin receptor-deficient *db/db* mice restored endothelial function, myogenic tone, and NO-dependent vascular regulation in mesenteric resistance arteries.^20^ Our study expands the role of NOX1 in metabolic disease from the peripheral to the myocardial vasculature, and from atherosclerosis and hypertension to the clinical entity of metabolic heart disease.

WT-HFHS hearts exhibited increased expression of the adhesion molecules VCAM-1 and ICAM-1 on endothelial cells, and higher numbers of Mac-2-, IL-1β-, and NLRP3-positive cells in the myocardial tissue. These inflammatory features were not observed in NOX1-deficient mice. Inflammation plays a key role in the pathogenesis of metabolic heart disease and HFpEF.^7,41^ Furthermore, IL-1 has been implicated in DD and clinical trials inhibiting IL-1 in HFpEF have been initiated.^42^ The precise mechanisms responsible for the lower inflammation seen in the heart in the absence of NOX1 remain to be established. Our data indicate that up to 24 weeks after STZ there were no differences in the frequencies of blood monocytes between WT- and KO-HFHS mice. Although we cannot exclude that monocyte mobilization may occur at a later time point in our model, our *in vitro* data suggest that the differences in the cardiac abundance of inflammatory cells may relate to an endothelium-dependent mechanism. Specifically, the lack of endothelial activation in NOX1-deficient mice may prevent immune cell recruitment to the heart, which is supported by the lower expression of ICAM-1 in NOX1-siRNA-treated endothelial cells and by previous observations of a role for NOX1 in oxidative stress, inflammation, and dysfunction in the extracardiac vasculature.^20,40^ Additionally, however, immune cell-intrinsic mechanisms in the absence of NOX1 may lead to impaired cell adhesion and transendothelial migration, or inhibit the expansion of cardiac resident immune cells. Although *Nox1* was not detectable in cardiac macrophages in the healthy heart, its expression might be induced under inflammatory conditions. *Nox1* was indeed expressed in BMDMs and upregulated in response to LPS, which is consistent with previous reports.^36^ Interestingly, we also found that *NOX1* was significantly higher expressed in peripheral monocytes from patients with DD compared to controls, although these data were retrieved from a small sample size of patients that were not diabetic. Still, whereas human DD and HFpEF present with many different phenotypes and are associated with various comorbidities, inflammation, endothelial dysfunction, and NO-depletion are known and common denominators of HFpEF in humans.^43,44^ Although our data indicate that NOX1 may be regulated in peripheral monocytes in pro-inflammatory conditions associated with DD and HFpEF in humans, further studies are needed to delineate the precise contribution of NOX1 in macrophages or other inflammatory cells in DD and HFpEF in general and in metabolic heart disease in particular.

Superoxide as produced primarily by NOXs reacts with NO to form the highly reactive peroxynitrite, which leads to nitration of protein tyrosine residues. We found increased nitrotyrosinylation at 44 weeks in the hearts of WT, but not of NOX1-deficient metabolic disease mice. This finding is consistent with the hypothesis that NOX1-derived superoxide leads to the consumption of NO through peroxynitrite formation and gives rise to the hypothesis that NO-depletion contributes to the cardiomyocyte hypertrophy in our model.

NOX1 could also mediate metabolic heart disease through regulation of vascular smooth muscle cells (VSMCs). In a mouse model of smooth muscle cell-specific deficiency of the NOX1 regulating subunit NOXA1, NOX1 contributed to VSMC activation, ROS production, and VCAM-1 expression in the aorta after endovascular injury and in atherogenesis.^45^ However, we found low expression of *NOX1* in HCASMC and no regulation in response to LPS, suggesting that NOX1 in VSMCs may not play a major role in our model.

Although our data support that NOX1-deficiency is sufficient to prevent endothelial activation and myocardial remodelling in a mouse model of metabolic heart disease and find NOX1 upregulated in peripheral monocytes of humans with DD due to other cause, it has to be pointed out that until now, no NOX1-targeting therapy is available for human use. In addition, potential side effects of systemic, non-isoform-specific NOX inhibition have to be kept in mind. Therefore, therapeutic targeting of NOX1 downstream signalling in cardiovascular and/or immune cells may offer a therapeutic alternative. Interestingly, indirect inhibition of NOX1 was recently suggested as a therapeutic strategy in the context of aging-related cardiac remodelling and HFpEF.^46^ This proposition was based on beneficial effects seen in aged mice deficient for G protein-coupled oestrogen receptor (GPER), a molecule that has previously been shown to act as a constitutive activator of NOX1.^47^ However, to translate these and our findings to human DD and HFpEF, further studies are needed to identify the roles of NOX1 and to also explore NOX1 up- and downstream signalling in different cell types in human disease.

### 4.1. Study limitations

Our model is based on the constitutive genetic deletion of *Nox1*. However, such a model may more closely reflect anticipated effects of the pharmacological inhibition of NOX1. Although our data support a central role of the cardiac microvasculature, macrovascular alterations may likewise play a role. Moderately decreased baseline blood pressure has been described in NOX1^y/-^ mice in some studies,^21^ but not in others.^48,49^ We did not observe significant differences in blood pressure at 44 weeks between WT and NOX1-deficient mice, nor increase in arterial pressure in metabolic disease, which is consistent with previous reports.^20,40^ Nevertheless, increased aortic stiffness evoked by obesity and metabolic disease^50^ and possibly ameliorated by NOX1-deficiency may contribute to the cardiac phenotype. Similarly, we cannot rule out differences in blood pressure in the early stages of metabolic disease. In the healthy mouse heart, *Nox1* mRNA was predominantly expressed in CD64^−^ immune cells rather than in cardiac CD11b^+^CD64^+^ immune cells. Whether NOX1 is upregulated and contributes to CD11b^+^CD64^+^ or other immune cell function in metabolic disease remains to be determined. Finally, human data are retrieved from only a small subset of patients that were not diabetic. Therefore, how precisely NOX1 expression in peripheral monocytes in humans is linked to cardiac disease and to diabetes still needs to be established. In this context, it is also important to point out that whereas our model reliably produced endothelial activation and myocardial remodelling in metabolic disease, it did not reproduce DD or HFpEF as seen in humans.

## 5. Conclusion

NOX1 is upregulated in peripheral monocytes in patients with DD and its genetic deletion prevents cardiac endothelial activation, inflammation, and remodelling in metabolic disease in mice. NOX1 or NOX1-dependent signalling could therefore qualify as potential therapeutic target for the treatment of metabolic heart disease or other pro-inflammatory disease states associated with DD.

## Supplementary material

Supplementary material is available at *Cardiovascular Research* online.

## Funding

This work was supported by a grant from the Swiss National Science Foundation (grant number 310030_144208 to G.M.K.), the Foundation for Cardiovascular Research, Basel, the Swiss Diabetes Foundation, and the Medical Division of the Margarete and Walter Lichtenstein Foundation, University of Basel, Switzerland (all to G.M.K.). S.H. was supported by funding from the European Union Commission’s Seventh Framework programme under grant agreement N° 305507 (HOMAGE), the ERA-Net-CVD project MacroERA, 01KL1706, and IMI2-CARDIATEAM (N° 821508) and funding from the Netherlands Cardiovascular Research Initiative CVON, an initiative with support of the Dutch Heart Foundation (Hartstichting), CVON2016-Early HFPEF, 2015-10, CVON She-PREDICTS, 2017-21, and CVON Arena-PRIME, 2017-18. Furthermore, S.H. acknowledges the support of FWO G091018N and FWO G0B5930N. The HELPFul case-cohort study primarily received funding from the Dutch Heart Foundation (Hartstichting) 2013T084 (Queen of Hearts). A comprehensive list of investigators involved in the Queen of Hearts consortium can be found at: http://www.queen-of-hearts.eu. E.L.R. was supported by a CVON-RECONNECT Talent program grant from the Dutch Heart Foundation (Hartstichting). G.B.V. was supported by the RECONNECT program with a grant of the Netherlands Cardiovascular Research Initiative CVON 2014-11 RECONNECT.

## Authors’ contributions

Overall conceptualization: L.X., O.P., and G.M.K.; experimental and human study design: L.X., M.B., G.V., S.C.A.d.J., H.M.d.R., S.H., and G.M.K.; data acquisition, analysis, and interpretation of data: all authors; manuscript drafting: L.X., M.B., and G.M.K.; revision for important content: all authors; final approval: all authors; supervision: G.M.K.

## Supplementary Material

cvab349_Supplementary_Data

## Data Availability

The data underlying this article are available on reasonable request to the corresponding author.
