# Supplementary material for: NOX1 mediates metabolic heart disease in mice and is upregulated in monocytes of humans with diastolic dysfunction
Source: Cardiovasc Res. 2021 Nov 26;118(14):2973–84. doi: 10.1093/cvr/cvab349 (PMC9648822; doi:10.1093/cvr/cvab349)
Supplement: cvab349_Supplementary_Data [file cvab349_supplementary_data.zip › Xu_Supplement_R_clean.docx]

**Supplemental Information**

**NOX1 mediates metabolic heart disease in mice and is upregulated in monocytes of humans with diastolic dysfunction**

Lifen Xu^1^, Melania Balzarolo^1^, Emma L. Robinson^2^, Vera Lorenz^1^, Giacomo Della Verde^1^, Lydia Joray^1^, Michika Mochizuki^1^, Beat A. Kaufmann^1,3^, Gideon Valstar^4,5^, Saskia C. A. de Jager^4^, Hester M. den Ruijter^4^, Stephane Heymans^6,7,8^, Otmar Pfister^1,3^ and Gabriela M. Kuster^1,3,*^

^1^ Department of Biomedicine, University Hospital Basel and University of Basel, Basel, Switzerland; ^2^ Department of Cardiology, Cardiovascular Research Institute Maastricht, Maastricht University, Maastricht, The Netherlands; ^3^ Department of Cardiology, University Hospital Basel, Basel, Switzerland; ^4^ Laboratory of Experimental Cardiology, University Medical Center Utrecht, Utrecht University, Utrecht, The Netherlands; ^5^ Julius Center for Health Sciences and Primary Care, University Medical Center Utrecht, Utrecht University, Utrecht, The Netherlands; ^6^ Centre for Molecular and Vascular Biology, KU Leuven, Herestraat 49, bus 911, 3000 Leuven, Belgium; ^7^ Department of Cardiology, Maastricht University, CARIM School for Cardiovascular Diseases, Universiteitssingel 50, 6229 ER Maastricht, The Netherlands; ^8^ ICIN-Netherlands Heart Institute, Holland Heart House, Moreelsepark 1, 3511 EP Utrecht, The Netherlands

***Correspondence:** Gabriela M. Kuster, M.D., Myocardial Research, Department of Biomedicine, University of Basel and University Hospital Basel, Hebelstrasse 20, 4031 Basel, Switzerland, Tel: ++41 61 328 77 36, Fax: ++41 61 265 45 98, Email: [Gabriela.Kuster@usb.ch](mailto:Gabriela.Kuster@usb.ch)

**Supplementary Tables and Figures**

***Supplemental Table 1***. ***Echocardiographic parameters at baseline and at 12, 24, 36, and 44 weeks after STZ or vehicle injection in mice***

|  | **HR** | **LVAW; d** | **LVID; d** | **LVPW; d** | **LVAW; s** | **LVID; s** | **LVPW; s** | **EF** | **E/A** | **E/E'** |
| --- | --- | --- | --- | --- | --- | --- | --- | --- | --- | --- |
|  | bpm | mm | mm | mm | mm | mm | mm | % |  |  |
| **Baseline** | | | | | | | | | | |
| **WT-CTD (n=16)** | 486 ± 12 | 0.75 ± 0.02 | 4.17 ± 0.06 | 0.61 ± 0.02 | 1.02 ± 0.03 | 3.17 ± 0.08 | 0.92 ± 0.02 | 48.08 ± 1.93 | 1.42 ± 0.05 | 23.50 ± 1.53 |
| **WT-HFHS (n=16)** | 496 ± 13 | 0.76 ± 0.03 | 4.14 ± 0.08 | 0.61 ± 0.02 | 1.07 ± 0.04 | 3.16 ± 0.08 | 0.87 ± 0.03 | 47.43 ± 1.90 | 1.39 ± 0.07 | 28.64 ± 2.78 |
| **KO-CTD (n=16)** | 489 ± 18 | 0.73 ± 0.01 | 4.24 ± 0.06 | 0.59 ± 0.02 | 1.03 ± 0.02 | 3.20 ± 0.08 | 0.88 ± 0.03 | 49.02 ± 1.66 | 1.48 ± 0.08 | 23.70 ± 1.47 |
| **KO-HFHS (n=17)** | 486 ± 9 | 0.78 ± 0.02 | 4.21 ± 0.06 | 0.68 ± 0.01 | 1.06 ± 0.03 | 3.31 ± 0.07 | 0.90 ± 0.02 | 43.67 ± 1.64 | 1.48 ± 0.06 | 25.78 ± 2.26 |
| **12 weeks** | | | | | | | | | | |
| **WT-CTD** | 481 ± 14 | 0.84 ± 0.02 | 4.41 ± 0.06 | 0.68 ± 0.02 | 1.16 ± 0.03 | 3.37 ± 0.07 | 0.98 ± 0.02 | 47.07 ± 1.68 | 1.37 ± 0.06 | 23.11 ± 1.55 |
| **WT-HFHS** | 478 ± 17 | 0.82 ± 0.02 | 4.31 ± 0.07 | 0.69 ± 0.02 | 1.13 ± 0.03 | 3.29 ± 0.07 | 1.00 ± 0.03 | 47.83 ± 1.65 | 1.45 ± 0.13 | 24.45 ± 1.87 |
| **KO-CTD** | 484 ± 15 | 0.83 ± 0.02 | 4.41 ± 0.06 | 0.67 ± 0.01 | 1.10 ± 0.02 | 3.41 ± 0.08 | 0.97 ± 0.03 | 45.87 ± 1.90 | 1.54 ± 0.06 | 24.21 ± 2.04 |
| **KO-HFHS** | 454 ± 11 | 0.80 ± 0.02 | 4.35 ± 0.04 | 0.70 ± 0.01 | 1.08 ± 0.03 | 3.37 ± 0.05 | 0.98 ± 0.01 | 45.63 ± 1.02 | 1.52 ± 0.06 | 23.66 ± 1.22 |
| **24 weeks** | | | | | | | | | | |
| **WT-CTD** | 519 ± 12 | 0.85 ± 0.02 | 4.60 ± 0.06 | 0.71 ± 0.01 | 1.16 ± 0.03 | 3.61 ± 0.07 | 0.95 ± 0.03 | 43.51 ± 1.49 | 1.41 ± 0.05 | 26.85 ± 2.14 |
| **WT-HFHS** | 512 ± 9 | 0.90 ± 0.01 | 4.42 ± 0.09 | 0.73 ± 0.02 | 1.23 ± 0.02 | 3.41 ± 0.09 | 1.01 ± 0.02 | 46.19 ± 1.54 | 1.35 ± 0.06 | 24.30 ± 1.79 |
| **KO-CTD** | 511 ± 12 | 0.87 ± 0.01 | 4.45 ± 0.08 | 0.69 ± 0.01 | 1.25 ± 0.03 | 3.37 ± 0.12 | 0.96 ± 0.03 | 48.28 ± 2.46 | 1.43 ± 0.06 | 21.46 ± 1.45 |
| **KO-HFHS** | 486 ± 12 | 0.88 ± 0.02 | 4.51 ± 0.04 | 0.74 ± 0.01 | 1.18 ± 0.03 | 3.55 ± 0.05 | 1.00 ± 0.02 | 43.50 ± 1.04 | 1.48 ± 0.08 | 21.87 ± 1.69 |
| **36 weeks** | | | | | | | | | | |
| **WT-CTD** | 510 ± 9 | 0.93 ± 0.02 | 4.56 ± 0.04 | 0.75 ± 0.02 | 1.28 ± 0.03 | 3.45 ± 0.07 | 1.04 ± 0.04 | 48.12 ± 1.78 | 1.33 ± 0.04 | 23.93 ± 2.64 |
| **WT-HFHS** | 534 ± 15 | 0.99 ± 0.03 | 4.50 ± 0.09 | 0.83 ± 0.02 | 1.34 ± 0.04 | 3.56 ± 0.07 | 1.08 ± 0.03 | 42.82 ± 1.15 | 1.37 ± 0.07 | 18.68 ± 1.71 |
| **KO-CTD** | 542 ± 8 | 0.92 ± 0.02 | 4.53 ± 0.07 | 0.75 ± 0.01 | 1.25 ± 0.02 | 3.54 ± 0.09 | 0.99 ± 0.03 | 44.45 ± 1.63 | 1.42 ± 0.06 | 22.67 ± 2.75 |
| **KO-HFHS** | 519 ± 9 | 0.93 ± 0.02 | 4.59 ± 0.05 | 0.79 ± 0.02 | 1.27 ± 0.03 | 3.57 ± 0.07 | 1.06 ± 0.02 | 44.81 ± 1.29 | 1.35 ± 0.06 | 20.65 ± 1.42 |
| **44 weeks** | | | | | | | | | | |
| **WT-CTD** | 541 ± 5 | 0.94 ± 0.02 | 4.58 ± 0.06 | 0.79 ± 0.02 | 1.29 ± 0.03 | 3.49 ± 0.06 | 1.09 ± 0.03 | 47.62 ± 1.33 | 1.28 ± 0.06 | 20.79 ± 1.49 |
| **WT-HFHS** | 513 ± 9 | 1.01 ± 0.03 | 4.61 ± 0.11 | 0.84 ± 0.02 | 1.36 ± 0.04 | 3.62 ± 0.10 | 1.11 ± 0.03 | 43.57 ± 1.75 | 1.49 ± 0.14 | 22.78 ± 1.98 |
| **KO-CTD** | 528 ± 13 | 0.92 ± 0.02 | 4.50 ± 0.07 | 0.75 ± 0.01 | 1.26 ± 0.04 | 3.44 ± 0.09 | 1.04 ± 0.03 | 47.46 ± 1.67 | 1.52 ± 0.05 | 21.66 ± 1.44 |
| **KO-HFHS** | 508 ± 7 | 0.91 ± 0.02 | 4.68 ± 0.06 | 0.80 ± 0.01 | 1.28 ± 0.03 | 3.70 ± 0.07 | 1.07 ± 0.03 | 42.97 ± 1.29 | 1.28 ± 0.05 | 21.55 ± 2.07 |
| **Statistics (linear mixed effect model)** | | | | | | | | | | |
| **WT-CTD vs. KO-CTD** | 1.000 | 0.999 | 0.254 | 0.996 | 0.972 | 0.970 | 0.883 | 0.867 | 0.761 | 0.996 |
| **WT-CTD vs. WT-HFHS** | 0.809 | 0.186 | 0.855 | **0.036*** | 0.734 | 0.322 | 0.564 | 0.215 | 0.445 | 0.256 |
| **KO-CTD vs. KO-HFHS** | 0.917 | 0.730 | **0.020*** | 0.699 | 0.999 | 0.554 | 0.864 | 0.963 | 0.296 | 0.842 |
| **WT-HFHS vs. KO-HFHS** | 0.987 | **0.018*** | 0.990 | **<0.001***** | 0.529 | 0.812 | 0.573 | 0.337 | 0.110 | 0.827 |

***Supplemental Table 2***. ***Left ventricular hemodynamic parameters at 44 weeks after STZ or vehicle injection in mice***

|  | **Heart Rate** | **Pes** | **dP/dt max** | **dP/dt min** | **Tau** | **ESPVR (Ees)** | **EDPVR (β)** |
| --- | --- | --- | --- | --- | --- | --- | --- |
|  | bpm | mmHg | mmHg/s | mmHg/s | ms |  |  |
| **WT-CTD (N=9)** | 492 ± 19 | 82.20 ± 4.74 | 7467 ± 961 | -7786 ± 1148 | 7.18 ± 0.55 | 7.88 ± 1.20 | 0.029 ± 0.010 |
| **WT-HFHS (N=10)** | 440 ± 15 | 79.09 ± 3.81 | 5477 ± 380 | -4646 ± 383 | 8.71 ± 0.49 | 4.99 ± 0.78 | 0.062 ± 0.014 |
| **KO-CTD (N=10)** | 508 ± 15 | 81.79 ± 3.32 | 6973 ± 509 | -7094 ± 538 | 7.07 ± 0.56 | 7.50 ± 1.55 | 0.024 ± 0.006 |
| **KO-HFHS (N=10)** | 460 ± 18 | 78.68 ± 2.47 | 6119 ± 390 | -5856 ± 605 | 7.76 ± 0.61 | 3.82 ± 0.85 | 0.041 ± 0.018 |
| **Statistics (2-way ANOVA)** | | | | | | | |
| **WT-CTD vs. KO-CTD** | 0.769 | 0.996 | 0.806 | 0.745 | 0.988 | 0.965 | 0.955 |
| **WT-CTD vs. WT-HFHS** | 0.071 | 0.799 | **0.046*** | **0.007**** | 0.120 | 0.161 | 0.157 |
| **KO-CTD vs. KO-HFHS** | 0.094 | 0.790 | 0.513 | 0.381 | 0.614 | 0.052 | 0.585 |
| **WT-HFHS vs. KO-HFHS** | 0.633 | 0.996 | 0.683 | 0.397 | 0.399 | 0.716 | 0.430 |

Pes: endsystolic pressure; ESPVR: end-systolic pressure volume relationship; Ees is the slope calculated from the linear line fits for ESPVR. EDPVR: end-diastolic pressure volume relationship; β is the coefficient from the exponential curve (P = Ce^βV^) fits for EDPVR.

***Supplemental Table 3***. ***Characteristics of the HELPFul case-control study patients***

|  | **Case Male** | **Case Female** | **Control Male** | **Control Female** |
| --- | --- | --- | --- | --- |
|  |  |  |  |  |
| n | 4 | 6 | 4 | 6 |
| Age (yrs) | 67.03 ± 14.05 | 64.77 ± 9.02 | 62.24 ± 12.8 | 62.45 ± 9.08 |
| HFpEF score [^1^](#_ENREF_1) | 5 | 5 | 0.25 | 0.5 |
| LVEF (%) | 66.75 ± 13.77 | 64.17 ± 8.98 | 62 ± 12.73 | 62.17 ± 8.93 |
| BMI | 28.15 ± 5.52 | 27.56 ± 5.26 | 26.64 +/- 2.27 | 23.05 ± 1.8 |
| BNP (pg/mL) | 129.55 ± 107.03 | 73.22 ± 19.06 | 13.23 ± 3.15 | 26.23 ± 19.02 |
| hsTnI (pg/mL) | 31.58 ± 39.63 | 6.83 ± 6.33 | 3.97 ± 1.33 | 2.77 ± 2.44 |
| Creatinine (umol/L) | 93.35 ± 23.60 | 56.48 ± 7.76 | 87.00 ± 13.98 | 59.52 ± 2.74 |
| hsCRP (mg/L) | 2.29 ± 1.33 | 4.36 ± 4.72 | 1.18 ± 1.07 | 2.28 ± 3.28 |
| CYSC (mg/L) | 1.20 ± 0.32 | 0.85 ± 0.19 | 1.02 ± 0.19 | 0.83 ± 0.1 |
| eGFRcys | 70.36 ± 20.59 | 92.67 ± 17.24 | 81.09 ± 16.02 | 93.14 ± 7.59 |
| Atrial Fibrillation (%) | 0 | 0 | 0 | 0 |
| Hypertension (%) | 25 | 50 | 25 | 50 |
| Diabetes (%) | 25 | 0 | 0 | 0 |
| Smoker, ever (%) | 25 | 50 | 25 | 66.7 |
| Smoker, current (%) | 25 | 0 | 0 | 0 |
| Dyslipidemia (%) | 25 | 16.67 | 50 | 33.33 |
| COPD (%) | 25 | 0 | 25 | 16.67 |

Case: patient identified as having diastolic dysfunction; COPD: chronic obstructive pulmonary disorder; eGFRcys: estimated glomerular filtration rate based on the CKD-EPI formula using creatinine and cystatin C levels [^2^](#_ENREF_2); BNP: Brain natriuretic peptide; sTnI: high sensitivity troponin I; hsCRP: high sensitivity C-reactive protein; CYSC: cystatin C. Continuous values are mean ± SD unless otherwise stated.

***Supplemental Figure S1***

***Supplemental Figure S1. Mouse genotyping.***  The 800 bp product corresponds to wild-type (WT) NOX1, the 340 bp product corresponds to the knock-out (KO). Lanes 1, 2, 3: WT male; lane 4: KO male (hemizygous), lane 5: WT male; lanes 6 and 7: heterozygous females.

***Supplemental Figure S2***

**
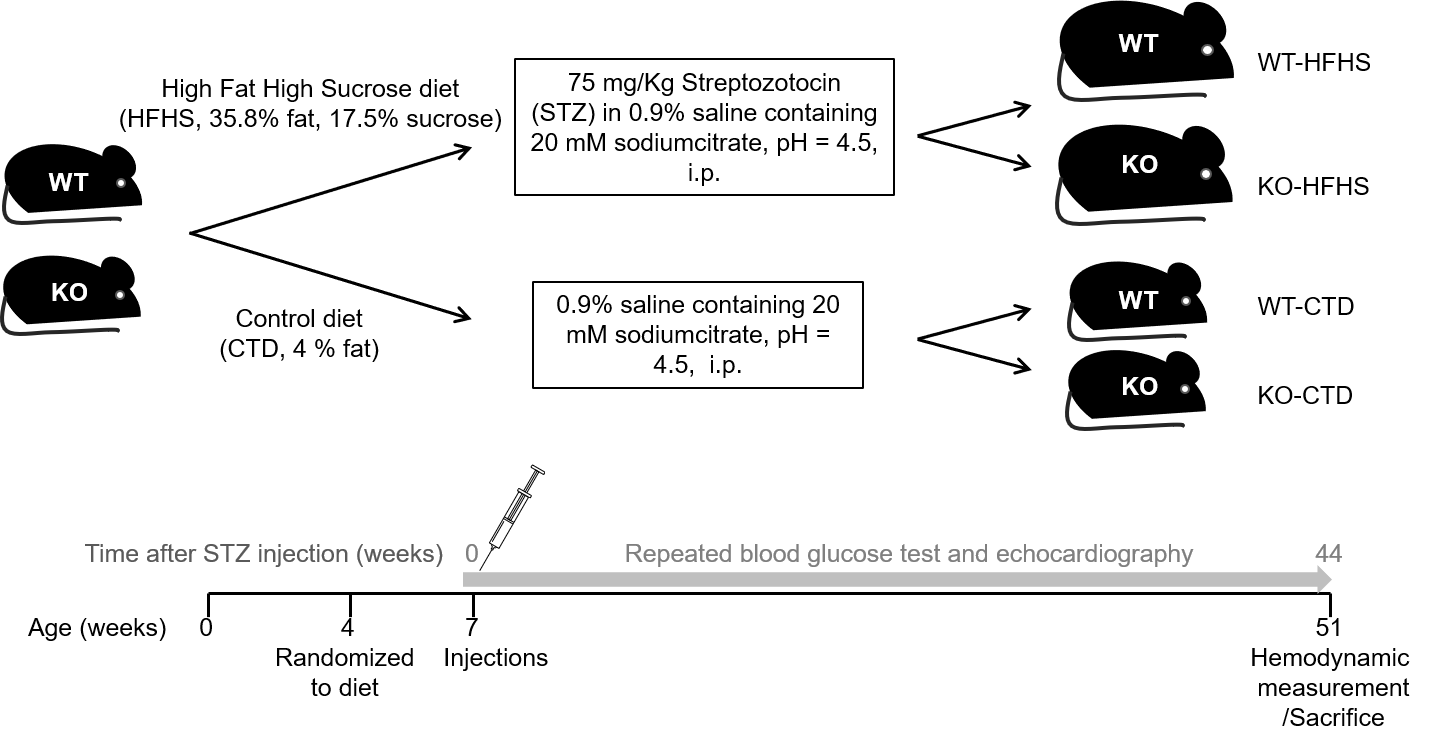
**

***Supplemental Figure S2. Experimental model of HFHS/STZ-induced metabolic disease.***  NOX1^y/-^ (KO) mice and male WT littermates were randomized to HFHS or CTD at 4 weeks of age and injected once with 75 mg/kg STZ or vehicle (0.9% saline containing 20 mM sodium citrate) after 3 weeks. Diet was continued for another 44 weeks. Blood glucose was tested at 2-4 week intervals and echocardiography was performed at 8-12 week intervals. After the last echocardiography, mice were either sacrificed immediately for organ collection or followed by hemodynamic measurement before sacrifice.

***Supplemental Figure S3***


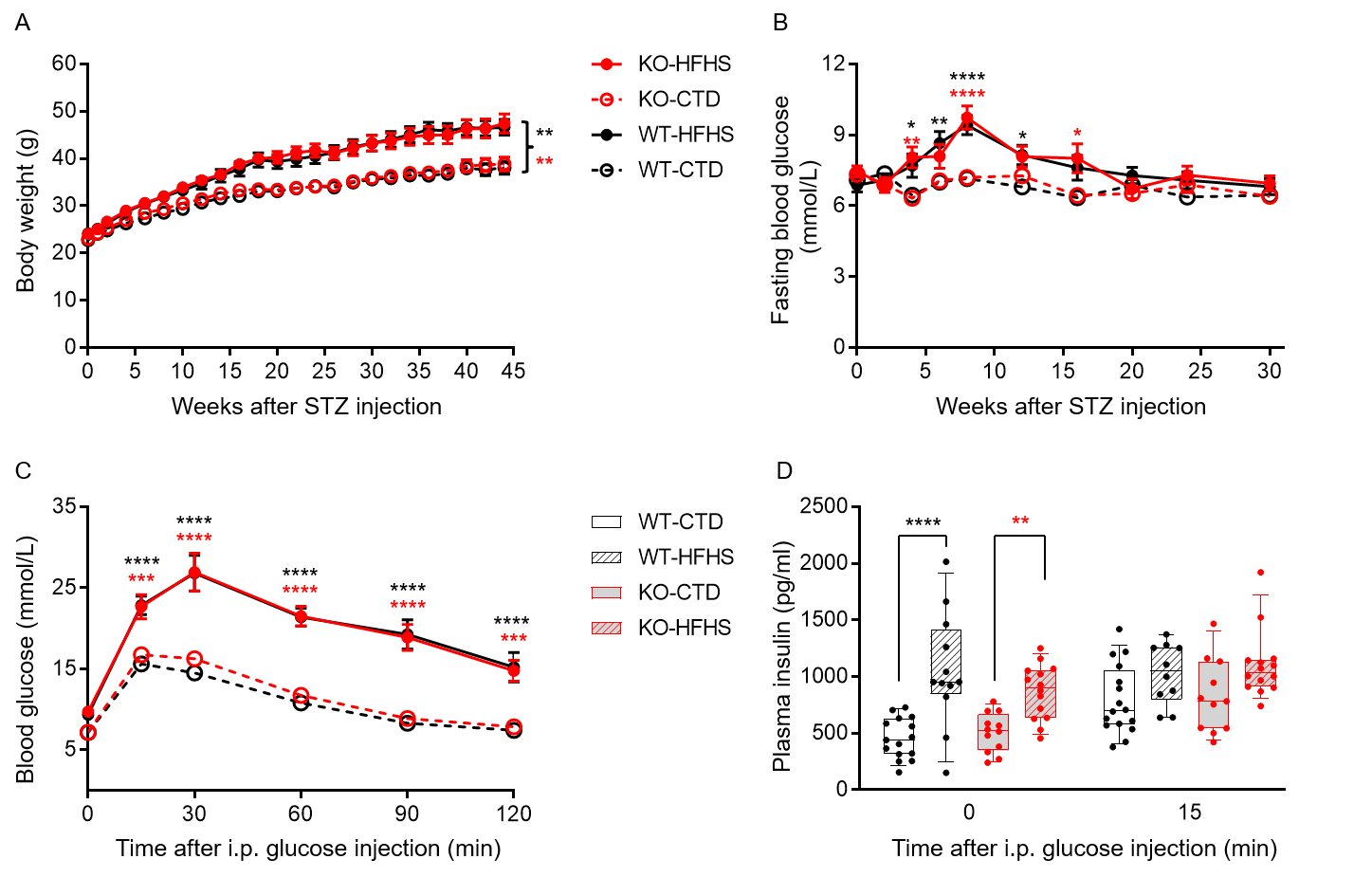


***Supplemental Figure S3. Weight gain, fasten blood glucose, glucose tolerance test and plasma insulin in wild-type and NOX1^y/-^ mice under normal diet and after vehicle injection versus high fat high sucrose diet and streptozotocin injection (HFHS/STZ).* (A)** Body weight gain over time. Groups were compared by fitting a RStudio linear mixed effect model: **p<0.01; **(B)** Fasting blood glucose, **(C)** glucose tolerance test and **(D)** plasma insulin at 8 weeks after STZ injection; groups were compared using two-way ANOVA with Sidak test at each time point, **p<0.01, ***p<0.001, ****p<0.0001, n=16 for WT-CTD, 16 for WT-HFHS, 16 for KO-CTD, 17 for KO-HFHS.

***Supplemental Figure S4***


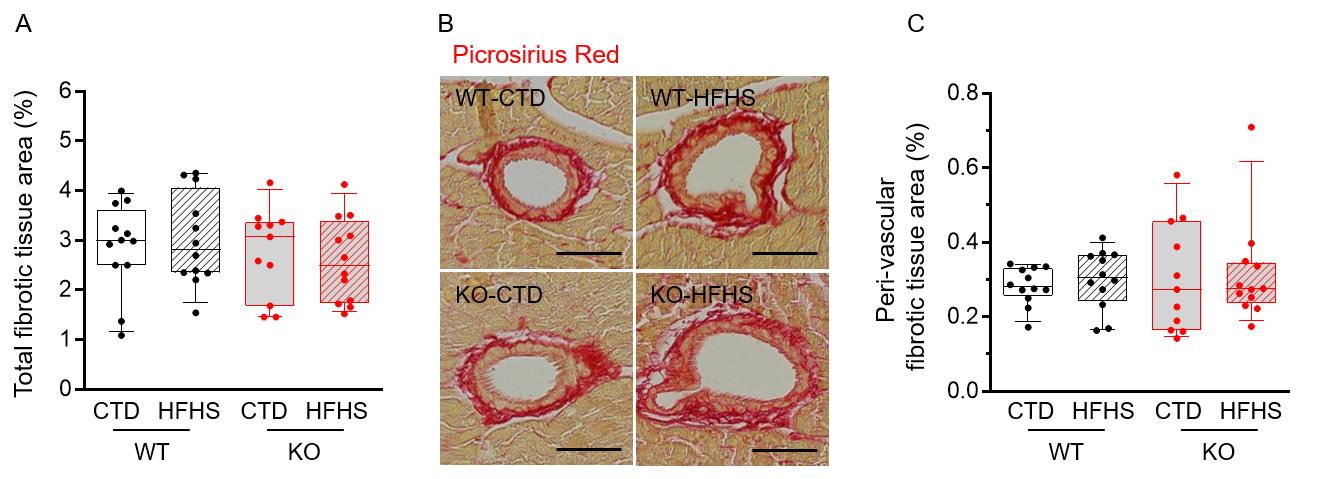


***Supplemental Figure S4. Fibrosis accessed by picrosirius red staining.*** **(A)** Quantification of total fibrotic tissue area as percentage to left-ventricular tissue area. **(B)** Representative photomicrographs of peri-vascular fibrosis. Scale bar: 50 μm **(C)** Quantification of fibrotic tissue area as percentage to left-ventricular tissue area; n=12 for WT-CTD, 12 for WT-HFHS, 11 for KO-CTD, 12 for KO-HFHS.

***Supplemental Figure S5***


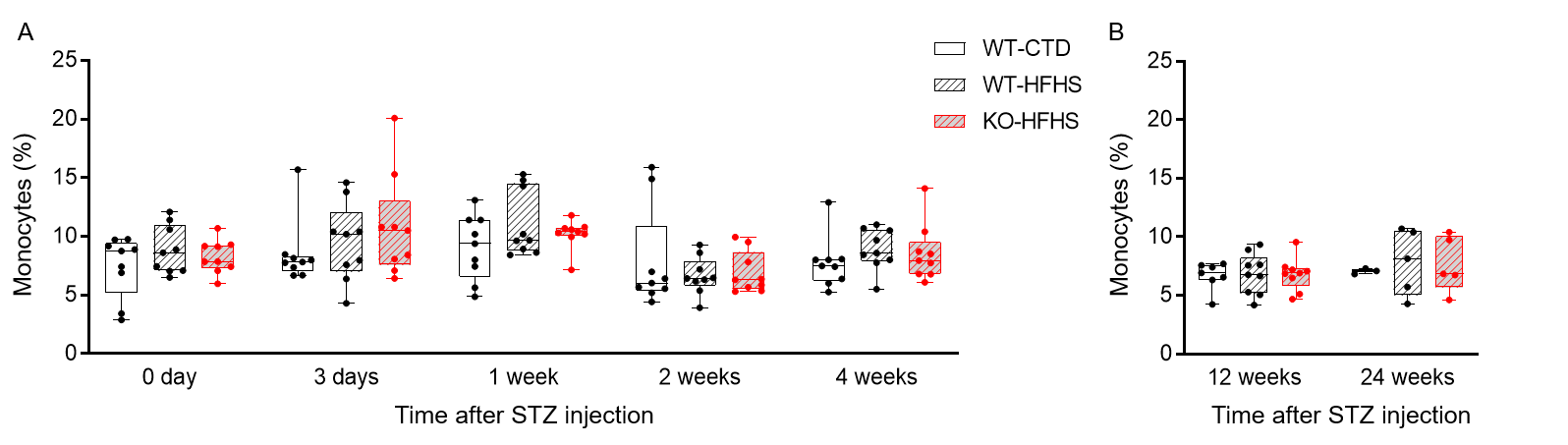


***Supplemental Figure S5. Percentage of monocytes in the peripheral blood as determined by FACS.* (A)** n=9 per group; **(B)** n=7 in CTD group and n=9 in HFHS groups at 12 weeks, n=3 in CTD group and n=5 in HFHS groups at 24 weeks.

***Supplemental Figure S6***


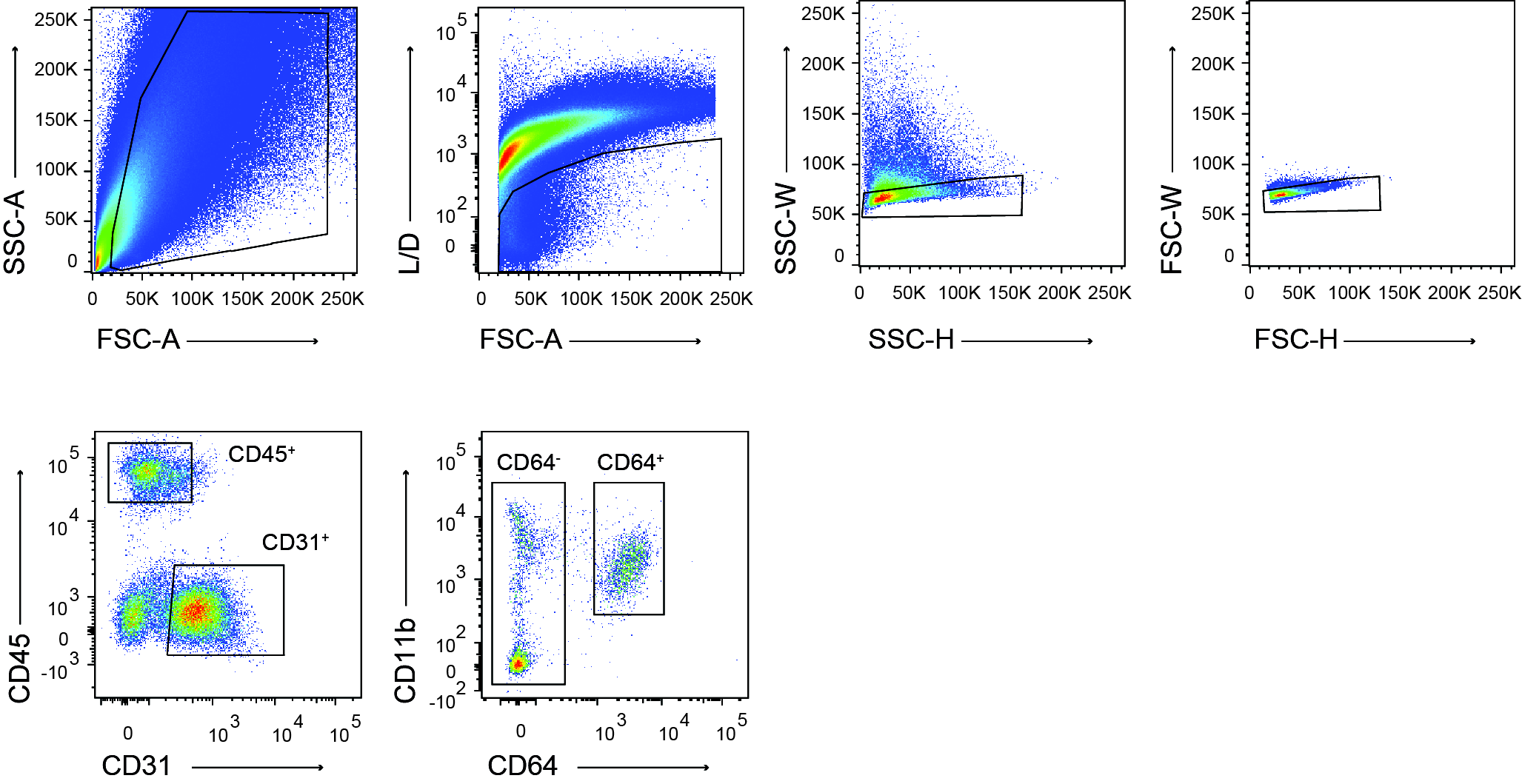


***Supplemental Figure S6.*** Flow cytometry plots representing the full gating strategy used for sorting of CD45^+^ and CD31^+^ and cells, and CD11b^+^CD64^+^ (CD64^+^) and CD64^-^ subpopulations from WT mouse hearts as in **Figure 4**. Plots in lower panel are the same as in **Figure 4A**. In sorting for **Figure 4B** doublet exclusion was performed after CD45^+^ cell gating.

***Supplemental Figure S7***

***
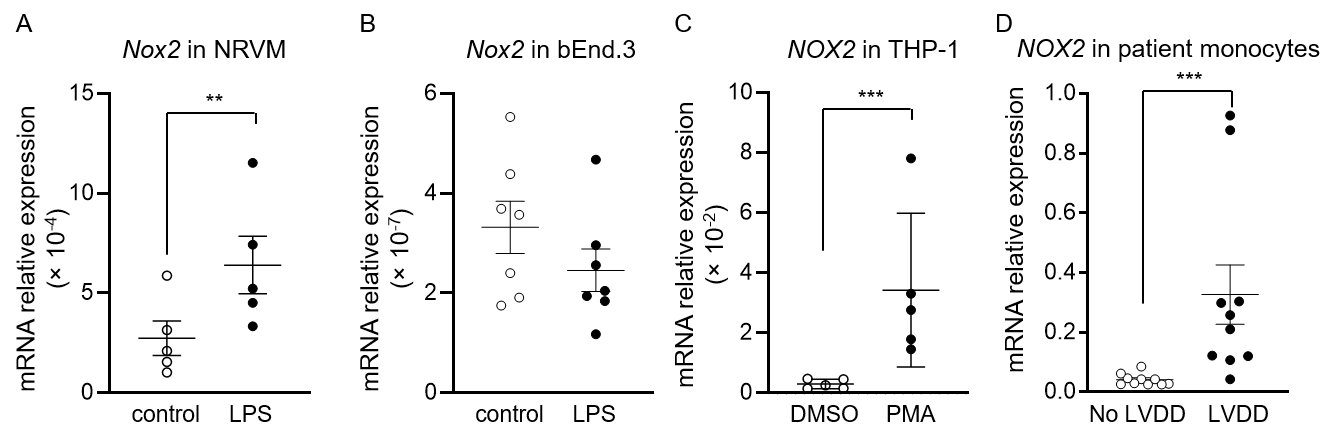
***

***Supplemental Figure S7.*** ***NOX2 expression at baseline and in response to LPS stimulation***. NOX2 mRNA levels were assessed in NRVM, bEnd.3 and THP-1 cells (in which regulation of NOX1 could be shown) at baseline and upon LPS or PMA stimulation as per Figure 4, and in human peripheral blood monocytes as per Figure 5A. (**A**-**C**) Groups were compared using ratio-paired t-test, **p<0.01, ***p<0.001. (**D**) Groups were compared using unpaired Mann-Whitney test.

***Supplemental Figure S8***

**
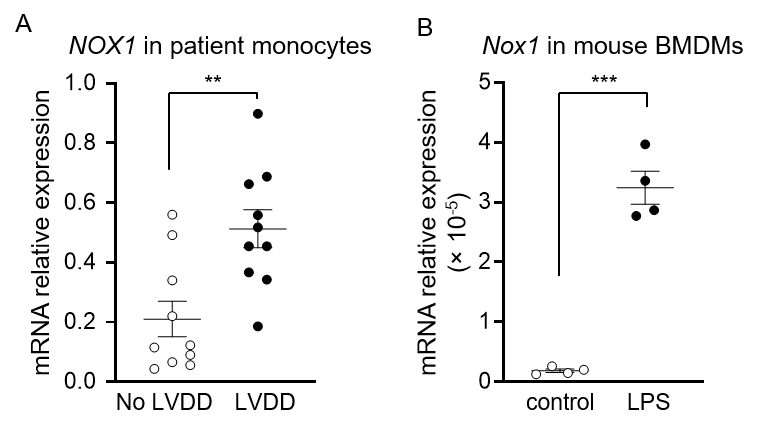
**

***Supplemental Figure S8. NOX1 expression in CD14^+^ peripheral blood monocytes from patients with DD and in BMDMs from mice.*** (**A**) CD14^+^ monocytes were sorted from isolated peripheral blood mononucleated cells from individuals from the HELPFul case-cohort study and *NOX1* mRNA levels were assessed by RT-qPCR (n=10 patients per group, unpaired Mann-Whitney test). (**B**) BMDMs from WT mice were stimulated with 100 ng/ml LPS or medium alone for 6 hours. Cells were collected and *Nox1* mRNA expression was assessed by RT-qPCR (n=4 mice, ratio paired t test). Data are shown as mean±SEM. **p<0.01, ***p<0.001. LVDD: left ventricular diastolic dysfunction.

**Supplementary Methods**

***Genotyping***

DNA was extracted from toes of 6-12 days old mice by digestion in 100 mM TrisHCl-Buffer pH 8.5 supplemented with 10 mg/ml Proteinase K (Thermo Scientific) at 56°C overnight. After centrifugation at 14800 rpm for 5 min the supernatant was heated up for 5 min at 95°C. PCR was performed using FIREPol Master Mix (Solis BioDyne) and the following Primers (Microsynth): Reverse primer for KO: 5’-TCG GAT CGA GCG CTC TGA AGT TCC T-3’; Reverse primer for WT: 5’-GGG ACA GCT TCC TGC ATC CCT CTG T-3’; Forward primer for both: 5’-TAG CCT GGC TGT TCC CTC ACC CAA A-3’, with the following PCR cycling program (Biometra Tadvanced): 94°C 2 min; 38 cycles 94°C 45 sec followed by 68°C 45 sec and 72°C 1 min; 72°C 10 min. After running a 2% agarose gel with RedSafe Staining Solution (Intron Biotechnology, #21141) the VILBER Fusion FX (Fusion FX7Edge software) was used to detect the bands. The size of the PCR product of 800 bp indicates WT, the PCR product of 340 bp indicates KO (**Supplemental Figure S1**).

***Fasting blood glucose and glucose tolerance test***

For glucose testing, mice were fasted for six hours (8:00am-2:00pm), prior to collection of 1-2 μl blood from the tail vein. Blood glucose concentration was determined by averaging two measurements obtained using a glucose meter (FreeStyle lite; Abbott Diabetes Care, Inc., Alameda, USA). For glucose tolerance testing (GTT), mice were fasted for six hours (8:00am-2:00pm) prior to injection of 2 g/kg glucose intraperitoneally. Blood glucose concentration was measured prior to injection (time 0) and 15, 30, 60, 90, and 120 min after. At time 0 and 15 min after glucose administration, blood (25 μl) was drawn from the tail vein into EDTA-containing Eppendorf tubes and maintained on ice for later plasma separation and insulin measurement.

## *Insulin concentration measurements*

Plasma insulin concentration was measured by electro-chemiluminescence using a mouse/rat insulin kit (K152BZC; Mesoscale Discovery, Rockville, USA), according to the manufacturer’s instructions.

***Histology***

Heart sections (4 μm thick and 450 μm interval) were cut at the mid-papillary muscle level using a Hyrax M55 microtome and mounted on microscopic glass slides for immunohistochemistry. Tissue slides were deparaffinized through sequential 5 min incubations in UltraClear (Biosystems), ethanol (100%, 96%, 70%) and water prior to further staining as described below.

***Wheat Germ Agglutinin (WGA) and Isolectin B4 (IB4) Staining***

WGA – IB4 staining was performed in deparaffinized formalin-fixed tissue slides using the lectin from triticum vulgaris (wheat) (WGA, L4895, Sigma, St. Louis, USA) and griffonia simplicifolia lectin I isolectin B4 (GSLI-IB4, DL-1207, Vector Laboratories, Inc. Burlingame, USA). Antigen retrieval was performed by incubating tissue sections in 97°C citrate-based antigen unmasking solution (Vector Laboratories, CA, USA; diluted 1:100 in distilled water) for 25 min. After blocking with 10% normal goat serum (50062Z, life technologies, Frederick, USA), sections were incubated with WGA (diluted 1:100 in 10% normal goat serum) and GSLI-IB4 (diluted at 1:25 in 10% normal goat serum) overnight at 4°C, followed by incubation with DAPI for 5 min at room temperature. Slides were subsequently washed and mounted with SlowFade Antifade Kit (S2828, Invitrogen, Eugene, USA). Microscopic images were taken with Olympus UPlanSApo 20×/0.75 objective on an Olympus BX61 Apollo microscope. Quantification was done with Fiji ImageJ software in a blinded fashion. For cardiomyocyte cross-sectional area measurement, only the cells with clear cross-sectional view and visibly centered nuclei were chosen. The membrane of individual cells was manually traced and selected as a ROI and the areas of these ROIs were measured. In average about 300 cells per heart were measured. For quantification of vessel density, myocardium with clear cross-sectional view of cardiomyocytes was selected as ROI in a rectangular or quadrilateral shape. The area of ROI was measured as A. The IB4 positive vessels within the ROI were counted as N_1_, the positive vessels on two of the ROI border sides were counted as N_2_. The vessel density was calculated based on the formula: vessel density = (N_1_ + N_2_) / A.

***Picrosirius Red Staining***

The deparaffinized and rehydrated tissue sections were immersed in picric acid saturated Sirius red solution for 1 h and washed briefly with water then re-dehydrated and mounted with UltraKitt mounting medium. The whole heart tissue was scanned with Nikon Plan APO 10×/0.45 objective or investigated with Nikon Plan APO 20×/0.75 objective on a Nikon Eclipse Ti microscope. Quantification was done on the scanned images with Fiji ImageJ software in a blinded fashion. In brief, the color channel of fibrosis and color channel of myocardium were separated from the RGB image by color deconvolution with the inbuilt ‘FastRed FastBlue DAB’ plugin. Threshold intensity was used to select the region of interest (ROI) on the two channels, respectively. The area of ROI was measured and the value was used to calculate the percentage of fibrotic tissue area to total myocardium area. Three sections per heart at mid-papillary muscle level were stained for investigation and one section per heart was quantified.

***Nitrotyrosine (NT3) Staining***

The deparaffinized and rehydrated tissue sections were incubated in 97°C citrate-based unmasking solution for antigen retrieval as described above. After 3% H_2_O_2_ and 3% BSA/PBS blockage, the sections were incubated with anti-NT3 (06-284, Millipore) antibody diluted at 1:150 in 1% BSA/PBS at 4°C overnight. A horseradish peroxidase (HRP) goat anti-rabbit immunoglobulin antibody combined with liquid 3,3’-diaminobenzidine (DAB) in substrate-chromogen system (Dako North America, Inc., Carpinteria, USA) was used for NT3 detection. The whole heart tissue was scanned with Nikon Plan APO 10×/0.45 objective or investigated with Nikon Plan APO 40×/0.75 objective on a Nikon Eclipse Ti microscope. Quantification was done on the scanned images with Fiji ImageJ software in a blinded fashion. In brief, the color channel of NT3 was separated from the RGB image by color deconvolution with the inbuilt ‘FastRed FastBlue DAB’ plugin. Threshold intensity was used to select the whole myocardium region as ROI. The mean grey intensity of ROI was measured as I. The optical density (OD) was calculated as OD = log(255/I). One section per heart at mid-papillary muscle level were stained for investigation and quantification.

***VCAM-1 and ICAM-1 Staining***

Deparaffinized and rehydrated heart sections were incubated with in 97°C Tris-EDTA buffer containing 10 mM Tris Base, 1 mM EDTA and 0.05% Tween-20 (pH = 9.0) for 30 min for antigen retrieval. After blockage with 3% H_2_O_2_ for 10 min and 3% BSA in PBS for 30 min, the tissues were incubated with rabbit anti-mouse/rat/human VCAM-1 antibody (clone EPR5047, Abcam, Cambridge UK) diluted 1:300 and purified rat anti-mouse CD54 antibody (ICAM-1, clone YN1/1.7.4, Biolegend) diluted 1:100 in 1% BSA/PBS at 4°C overnight. A horseradish peroxidase (HRP) goat anti-rabbit and goat anti-rat immunoglobulins antibody combined with liquid 3,3’-diaminobenzidine (DAB) in substrate-chromogen system (Dako North America, Inc., Carpinteria, USA) was used to detect the VCAM-1 and ICAM-1 antibody, respectively. Microscopic images were taken with Nikon CFI Plan APO Lambda 40×/0.95 objective on a Nikon Ti2 microscope. One section per heart at mid-papillary muscle level was investigated. Quantification was done with Fiji ImageJ software in a blinded fashion. In brief, the endothelium region was manually traced and the area was measured as A1. Color channel of VCAM-1 or ICAM-1 was separated from the RGB image by color deconvolution with the inbuilt ‘FastRed FastBlue DAB’ plugin. Threshold intensity was used to select ROI on the red channel within the endothelium region. The area of ROI was measured as A2. The percentage of VCAM-1 or ICAM-1 positive area was calculated as 100% × (A2 / A1). In average 6-7 arteries and 9-15 venules per heart were analyzed.

***Immunohistochemistry for Mac-2, NLRP3 and IL-1β***

Deparaffinized and rehydrated slides were incubated at 97°C for 25 minutes with the following antigen retrieval buffers: Citrate-based antigen unmasking solution (Vector Laboratories, CA, USA; diluted 1:100 in distilled water) for Mac-2 staining, and Tris-EDTA buffer (10 mM Tris Base, 1 mM EDTA and 0.05% Tween-20, pH = 9.0) for NLRP3 and IL-1β stainings. Sections were blocked with PBS containing 3% BSA and incubated at 4°C overnight with purified anti-mouse/human Mac-2 (M3/38, Biolegend, San Diego), rabbit polyclonal anti-mouse/rat/human NLRP3 or IL-1β antibodies (Abcam, Cambridge, UK) (diluted 1:100 in PBS/1% BSA). Incubation with goat anti-rat IgG Alexa 568 (for Mac-2, Life Technologies Europe BV) and goat anti-rabbit IgG Alexa 488 (for NLRP3 and IL-1β, Life Technologies Europe BV) secondary antibody was performed for 1 h at room temperature, followed by staining with DAPI for 10 min at room temperature. Slides were subsequently washed and mounted with home-made antifading medium (polyvinyl alcohol mounting medium with DABCO). The whole stained cardiac tissue was scanned with Nikon Plan APO 20×/0.75 objective on a Nikon Eclipse Ti fluorescent microscope. Quantification was done by manually counting the positive cells with Fiji ImageJ software in a blinded fashion regarding strain and diet/treatment allocation. Results were shown as number of positive cells / heart section. Mac-2 positive cells were the averages from three heart sections with about 450 μm intervals at mid-papillary muscle level. One section at mid-papillary muscle level was scanned for NLRP3 and IL-1β quantification.

***Tissue processing for flow cytometry***

After sacrifice mice were perfused with 15 ml ice-cold PBS and hearts were excised from the chest cavity. The atria and right ventricle were removed, and the left ventricle was washed in PBS, minced and digested with Collagenase B (1mg/ml, Roche) for 20 min at 37°C. The digested suspension was filtered through a 100 μm cell strainer, washed and incubated with red blood cell lysis buffer (155 mM NH_4_Cl, 10 mM KHCO_3_, 0,1 mM EDTA, pH 7.3) for 5 min on ice. Cells were subsequently filtered through a 40 μm cell strainer and washed with PBS, and stained for flow cytometry sorting. Blood was collected from the mouse tail vein, red blood cells were lysed, and cells were stained for further flow cytometry analysis.

***Flow cytometry analysis and sorting***

The following antibodies were used for flow cytometry analysis and sorting: anti-mouse CD45-Pacific Blue (clone 30-F11, BioLegend), anti-mouse CD31-APC (clone MEC 13.3, BD Pharmingen), anti-mouse/human CD11b-AF488 (clone M1/70, BioLegend) anti-mouse CD64-PE (clone X54-5/7.1, BioLegend) and anti-CD115-APC (clone AFS98, Biolegend). Stainings included discrimination of live/dead events using the Live/Dead Fixable Near-IR Dead Cell Staining Kit (Invitrogen) and were performed by incubation in the dark on ice for 30-50 min. Incubation with anti-mouse CD16/32 antibody solution (TruStain FcX, Biolegend) on ice for 15 min was performed prior to stainings to prevent unspecific antibody binding. Flow cytometry analysis and sorting were performed on a BD LSRFortessa analyzer and a BD FACSAria III sorter (BD Biosciences), respectively. Sorted cell populations from multiple hearts were pooled prior to further analysis. Short term changes in monocyte frequencies (up to 4 weeks) were assessed by incubating tail vein blood with anti-mouse CD16/32 antibody solution for 2 min followed by addition of antibodies and incubation in the dark on ice for 30 min. Red blood cells were then lysed and remaining cells were washed and resuspended for analysis on CytoFLEX flow cytometer (Beckman Coulter). Flow cytometry data analysis was performed with FlowJo software (Tree Star, Inc.)

***Generation and in vitro stimulation of murine bone marrow-derived macrophages***

Murine bone marrow-derived macrophages (BMDMs) were differentiated *in vitro* from murine bone marrow cells. Specifically, mouse tibias and femurs were resected from mice, bone marrow cells were flushed, filtered through a 70 μm cell strainer and washed with PBS. Red blood cells were lysed with lysis buffer (155 mM NH_4_Cl, 10 mM KHCO_3_, 0,1 mM EDTA, pH 7.3) for 5 min on ice, cells were washed and cultured in 100 mm non-tissue culture plates (2 × 10^6^ cell/plate) in complete medium (RPMI Medium 1640, HEPES (Gibco), 10% FCS, L-Glutamine, and Penicillin-Streptomycin) with 10 ng/ml murine M-CSF (Peprotech) for 8-9 days. Differentiation of BMDMs was assessed by CD11b and F4/80 staining. Upon differentiation cells were collected, seeded overnight in non-tissue culture-treated 12-well plates in serum free complete medium at a density of 400’000 cells/well and incubated the following day with 100 ng/ml LPS (Lipopolysaccharides from Escherichia coli O26:B6, Sigma-Aldrich) or serum-free medium for 6 hrs prior to collection for further analysis.

***Isolation of neonatal rat ventricular myocytes (NRVM) and cell culturing***

For NRVM isolation, hearts from 1 to 3-day old pups (Sprague Dawley) were digested in 0.05% Trypsin-EDTA (Gibco) followed by serial digestions in 0.07% (w/v) collagenase type II (Worthington) and pre-plated twice in T75 culture flasks (Sarstedt) to remove fibroblasts. Cardiomyocytes were seeded at a density of 1000 cells/mm^2^ on plastic culture dishes (Corning) and kept at 37˚C in Dulbecco's modified Eagle's medium (DMEM, Gibco) supplemented with 7% (v/v) heat-inactivated fetal calf serum (FCS), penicillin (P, 100 U/mL, Gibco), streptomycin (S, 100 µg/mL, Gibco) and 5-bromo-2-deoxyuridine (BrdU, 100 μmol/L, Sigma) for 24 hrs. Prior to treatments, the medium was changed to serum-free DMEM for 16 hrs. Mouse brain microvascular endothelial cells (bEnd.3 cells) (ATCC) were cultured in high glucose DMEM (Gibco) containing NEAA supplemented with L-Glutamine (2mM, Gibco), sodium pyruvate (1%, Gibco) and 10% FCS. Prior to treatments, the medium was changed to serum-free DMEM for 16 hrs. The human monocytic leukaemia cell line THP-1 (ECACC) was cultured in RPMI1640 (Gibco) supplemented with penicillin (P, 100 U/mL, Gibco), streptomycin (S, 100 µg/mL, Gibco) and 10% FCS. Human coronary artery smooth muscle cells (HCASMC) (Cell Applications) were cultured in smooth muscle cell growth medium (Cell Applications). All cells were cultured at 37°C in a humidified incubator with 5% CO_2_. bEnd.3 cells and HCASMC were treated with 100 ng/ml LPS (Sigma-Aldrich) for 6 hrs. THP-1 cells were treated with 100 ng/ml phorbol 12-myristate 13-acetate (PMA) (Sigma-Aldrich) for 48 hrs followed by 24 hrs resting in PMA-free medium.

***RNA interference***

siRNA targeting NOX1 was obtained from Qiagen (SI00240758) and all stars negative control (1027281, Qiagen) served as a negative control. 1×10^5^ bEnd.3 cells were plated on 60 mm plastic culture dishes (Corning). After 24 hrs cells were transfected with siRNA when reaching 50-60% confluence, according to the manufacturer’s protocol. Briefly, NOX1-specific siRNA or negative control were diluted in 150 µl serum free medium and mixed with 9 µl of Lipofectamine RNAiMAX Transfection Reagent (Invitrogen) pre-diluted in 150 µl serum free medium. After 15 min incubation at room temperature, the complexes were added to the cells in a final volume of 3 ml culture medium with a final concentration of 40 nM siRNA. Medium was changed after 6 hrs to fresh culture medium. After 24 hrs the medium was changed to serum-free medium for 16 hrs followed by LPS-treatment. The RNAi results were evaluated by quantitative real-time PCR (RT-qPCR).

***RNA isolation and RT-qPCR***

Cells obtained from sorting and *in vitro* cultures were washed with PBS and diluted in TRI Reagent (Sigma-Aldrich) or Extraction Buffer (PicoPure RNA Isolation Kit Arcturus, Applied Biosystems), and kept at -70°C until RNA isolation. RNA was isolated according to the TRI Reagent (Sigma-Aldrich) or PicoPure RNA isolation Kit Arcturus (Applied Biosystems) manufacturer’s protocol. cDNA was synthesized with the GoScript Reverse Transcription Mix, Random Primers (Promega), according to the manufacturer’s protocol. qPCR was performed with the 7500 Fast Real-Time PCR System (Applied Biosystems), with the GoTaq qPCR Master Mix (Promega) and the following primers (forward, reverse):

Rat *Nox1* (5’-GAG CCA CTG ACG TCC TGA CA-3’), (5’-AAA CCC CCA CCA CAG ACT TG-3’); *Nox2* (5’- CCA TTC GGA GGT CTT ACT TTG AAG-3’), (5’-CTG TCC GAA GTC TGT CCA CGT AC-3’); mouse *Nox1* (5’-CTG TCC TTC TTG AGG GGC AC-3’), (5’-GGC TTC TTC TGT AGC GTT CGA A-3’); *Nox2* (5’-TGT GGC TGT GAT AAG CAG GAG TT-3’), (5’-CCA AAG GGT CCA TCA ACT GCT A-3’); *Icam-1* (5’- CTG GAG ACG CAG AGG ACC TTA A-3’), (5’-CAC ACT TCA CAG TTA CTT GGC TCC-3’); human *NOX1* (5’-TGG ATG CCT TCC TGA AAT ATG A-3’), (5’-ATT CAA GCA GAG AGC AGA CGC-3’); *NOX2* (5’-AGG AGT TTC AAG ATG CGT GGA ), (5’-GTG ACC CCA ATC CCT GCT C-3’); *18s* (5’-CCA TTC GAA CGT CTG CCC TAT-3’), (5’-GTC ACC CGT GGT CAC CAT G-3’) was used as reference gene. mRNA relative expression is shown as 2^-dCT^.

***Human CD14^+^ circulating monocyte isolation***

First, peripheral blood mononuclear cells (PBMCs) were isolated from 20 ml whole blood, followed by flow cytometric sorting of CD14^+^ monocytes as previously described [^3^](#_ENREF_3). Briefly, PBMCs were isolated by Ficoll gradient fractionation, re-suspended in 1 ml of sterile, serum‐free cell freezing medium with DMSO (Sigma Aldrich). PBMCs were slowly frozen overnight at −80°C using a Nalgene freezing container (Sigma Aldrich) and stored in liquid nitrogen until further analyses were performed [^4^](#_ENREF_4). After gentle thawing from liquid nitrogen, PBMCs were washed with RPMI media 1640 (Gibco) supplemented with GlutaMax (Gibco) containing 25 nM HEPES, 1% penicillin/streptomycin and 2% Fetal Bovine Serum (FBS) (#10270–106, Gibco). PBMCs were gently filtered over a 40 μm cell strainer (Greiner bio-one), washed with RPMI media again and centrifuged at 350 g for 5 min at 4°C. Subsequently, the PBMCs were washed with cold PBS supplemented with 2% FBS and 20 mM EDTA, centrifuged at 350 g for 5 min at 4°C and re-suspended in cold PBS with 1% BSA. Then, PBMCs were incubated with 5 µl anti-CD14 antibody (RPE/Cy5 conjugated, clone RMO52, Beckman Coulter) for 30 min at room temperature in the dark, washed with cold PBS and centrifuged at 350 g for 5 min at 4°C. Dead cells were excluded from flow cytometric analysis and by staining cells with Fixable Viability Dye eFluor™ 506 (eBioscience) for 30 min at room temperature in the dark. Then, cells were washed with cold PBS with 1% BSA then loaded onto a MoFlo Astrios Cell Sorter (Beckman Coulter) for flow cytometric sorting. Gating analyses of the flow cytometry data were performed using Kaluza 1.3 software. We selected viable classical CD14^+^ monocytes that were negative for CD20, CD8 and CD4 (sorted separately; antibodies used anti-CD4-RPE/Cy7, anti-CD8-APC/A750, both Beckman Coulter and anti-CD20-A488, eBioscience) based on the combination of forward - sideward scatter. Isotype controls were used for each. Isolated CD14^+^ cells were re-suspended in 1 ml QIAzol lysis reagent (Ambion) before storing at – 80°C until further use.

***Gene expression analysis of human samples***

Total RNA was isolated from CD14^+^ monocytes by phenol-chloroform extraction according to the manufacturer’s instructions. RNA purity assessment and quantification was performed using a NanoDrop™ 2000/2000c (Thermo Scientific). cDNA synthesis was performed using 150 ng RNA and SuperScript™ III Reverse Transcriptase according to manufacturer’s instructions. The final cDNA reaction was diluted 1:10 in molecular biology grade water (Ambion, Thermo Scientific). QPCR was performed in 6 µl reactions in 384-well format on a LightCycler® 480 Instrument II (Roche) using iQ™ SYBR® Green supermix (BIO-RAD) with 1.6 µl cDNA and 200 nM concentration of each primer. Gene expression was normalized to the geometric mean of GAPDH, YWHAZ and RPL32, as selected from a panel of seven reference genes that were determined to be stable under the conditions examined [^5^](#_ENREF_5). The following primers purchased from Eurogentec were used (forward, reverse): *NOX1* (5’-GGT TTT ACC GCT CCC AGC AGA A-3’), (5’-CTT CCA TGC TGA AGC CAC GCT T-3’); *NOX2* (5’-CTC TGA ACT TGG AGA CAG GCA AA-3’), (5’-CAC AGC GTG ATG ACA ACT CCA G-3’); and the following reference genes: *YWHAZ* (5’-CGA GAT CCA GGG ACA GAG TC-3’), (5’-GGA TGT TCT GTG TCC GGA GT-3’); *GAPDH* (5’-ACC CAC TCC TCC ACC TTT GAC-3’), (5’- ACC CTG TTG CTG TAG CCA AAT T-3’); *RPL32* (5’-AGG CAT TGA CAA CAG GGT TC-3’), (5’-GAC GTT GTG GAC CAG GAA CT-3’).

**Supplemental References**

1. Pieske B, Tschope C, de Boer RA, Fraser AG, Anker SD, Donal E, Edelmann F, Fu M, Guazzi M, Lam CSP, Lancellotti P, Melenovsky V, Morris DA, Nagel E, Pieske-Kraigher E, Ponikowski P, Solomon SD, Vasan RS, Rutten FH, Voors AA, Ruschitzka F, Paulus WJ, Seferovic P, Filippatos G. How to diagnose heart failure with preserved ejection fraction: the HFA-PEFF diagnostic algorithm: a consensus recommendation from the Heart Failure Association (HFA) of the European Society of Cardiology (ESC). Eur Heart J 2019;**40**(40):3297-3317.

2. Levey AS, Stevens LA, Schmid CH, Zhang YL, Castro AF, 3rd, Feldman HI, Kusek JW, Eggers P, Van Lente F, Greene T, Coresh J, Ckd EPI. A new equation to estimate glomerular filtration rate. Ann Intern Med 2009;**150**(9):604-12.

3. Meeuwsen JAL, de Vries JJ, van Duijvenvoorde A, van der Velden S, van der Laan SW, van Koeverden ID, van de Weg SM, de Borst GJ, de Winther MPJ, Kuiper J, Pasterkamp G, Hoefer IE, de Jager SCA, Queen of Hearts C. Circulating CD14(+)CD16(-) classical monocytes do not associate with a vulnerable plaque phenotype, and do not predict secondary events in severe atherosclerotic patients. J Mol Cell Cardiol 2019;**127**:260-269.

4. Meeuwsen JAL, van Duijvenvoorde A, Gohar A, Kozma MO, van de Weg SM, Gijsberts CM, Haitjema S, Bjorkbacka H, Fredrikson GN, de Borst GJ, den Ruijter HM, Pasterkamp G, Binder CJ, Hoefer IE, de Jager SCA. High Levels of (Un)Switched Memory B Cells Are Associated With Better Outcome in Patients With Advanced Atherosclerotic Disease. J Am Heart Assoc 2017;**6**(9).

5. Vandesompele J, De Preter K, Pattyn F, Poppe B, Van Roy N, De Paepe A, Speleman F. Accurate normalization of real-time quantitative RT-PCR data by geometric averaging of multiple internal control genes. Genome Biol 2002;**3**(7):RESEARCH0034.
